# Identification and characterization of the HSP gene family in the Chinese giant salamander: Expression patterns under combined environmental stress

**DOI:** 10.1186/s12864-026-13091-1

**Published:** 2026-06-24

**Authors:** Mei Xie, Cheng Wang, Jingfang Li, Yanhui Yin, Qilan Gao, Yixing Xie, Fangpeng Zhang, Zhiyong Deng, Ying Wei, Wansheng Jiang

**Affiliations:** 1 Hunan Engineering Laboratory for Giant Salamander Resource Conservation and Comprehensive Utilization, School of Life Sciences, Jishou University, Zhangjiajie, Hunan 427000 China; 2 Key Laboratory of Hunan Forest Products and Chemical Industry Engineering, National and Local United Engineering Laboratory of Integrative Utilization Technology of Eucommia Ulmoides, Jishou University, Zhangjiajie, Hunan 427000 China; 3 Hunan Zhangjiajie Giant Salamander National Nature Reserve Affairs Center, Zhangjiajie, Hunan 427400 China; 4 Zhangjiajie Institute of Aeronautical Engineering, Zhangjiajie, Hunan 427000 China; 5 College of Wildlife and Protected Area, Northeast Forestry University, Harbin, Heilongjiang, 150040 China

**Keywords:** Chinese giant salamander, Heat shock protein, Gene family, Environmental stress, Gene expression

## Abstract

**Background:**

The Chinese giant salamander (*Andrias davidianus*) is a critically endangered living fossil species that is highly sensitive to changes in water temperature. However, systematic studies on the heat shock protein (HSP) gene family and its response mechanisms to environmental stress in this species remain limited. This study utilized transcriptome data from captive-bred salamanders exposed to combined temperature and pathogen stress. Bioinformatics tools were employed to identify the HSP gene family of *A. davidianus* (*AndHSP*) and to analyze their evolution, structure, and function, thereby revealing their regulatory mechanisms in response to environmental stress.

**Results:**

A total of 72 *AndHSPs* were identified and classified into five subfamilies. Phylogenetic analysis revealed that each subfamily is evolutionarily conserved and functionally related. Gene expression analysis demonstrated that pathogen infection induced the expression of *AndHSPs*, and elevated temperature significantly intensified this response. Nine key differentially expressed genes were identified, predominantly from the *AndHSP70* subfamily, with *AndHSP70-18* exhibiting rapid heat-induced expression. Tissue-specific analysis showed high expression of *AndHSP60* in the spleen. A qPCR validation confirmed the reliability of the transcriptome expression results.

**Conclusions:**

This study presents the first systematic identification of the *AndHSP* gene family and elucidates its cooperative stress response mechanisms under combined temperature and pathogen stress. These findings provide a molecular basis for understanding the species’ environmental adaptation and have important implications for its conservation and artificial breeding.

**Supplementary Information:**

The online version contains supplementary material available at https://doi.org/10.1186/s12864-026-13091-1.

## Background

Animals initiate stress responses when environmental conditions change abruptly, enabling them to resist stressors and restore homeostasis. Stress responses in animals can be categorized into four main types based on the nature of the stressor: physical, chemical, and biological stress, which arise from the natural environment, and anthropogenic stress, resulting from human activities. Changes in key environmental factors such as temperature, dissolved oxygen, and salinity can directly or indirectly affect the physiology, behavior, and survival of aquatic animals [1, 2]. In the context of global warming, changes in water temperature have become a critical factor leading to physiological disorders, population declines, and degradation of ecosystem services in aquatic organisms. When temperature exceeds the optimal range of a species, it triggers stress responses at physiological, biochemical, and behavioral levels [3–5]. Moreover, temperature stress often interacts with other stressors, producing complex combined effects [6]. Although the impact of global climate change on aquatic organisms may sometimes be gradual, a thorough investigation of their responses to temperature stress remains essential for assessing their sensitivity to climate change and for ensuring their future sustainable conservation in the face of extreme weather events.

Heat shock proteins (HSPs), also known as heat stress proteins, are a highly conserved class of proteins found in both prokaryotes and eukaryotes. They play a crucial role in maintaining cellular protein homeostasis and protecting cells from stress-induced damage. HSPs are typically classified by their molecular weight into subfamilies such as HSP90, HSP70, HSP60, HSP40, and small heat shock proteins (sHSPs). This classification system is widely used due to its simplicity and intuitiveness, facilitating the systematic development of functional and mechanistic studies [7]. Based on expression patterns, HSPs can be divided into two categories: constitutive HSPs, which are continuously expressed under normal conditions to maintain basal protein homeostasis and cellular function, and inducible HSPs, which are rapidly activated in response to physical, chemical, or biological stressors such as sudden temperature changes, pathogen infection, or hypoxia. Inducible HSPs are key components of the cellular stress defense system. For example, under heat stress, the mRNA levels of the constitutively expressed *Ma-HSC70* and the inducibly expressed *Ma-HSP70* genes in *Megalobrama amblycephala* Yih, 1955 differ significantly, reflecting the specificity in structure, function, and regulation among different members of the HSP70 subfamily [8, 9]. Moreover, HSPs can be further subdivided based on their biological functions (e.g., chaperone activity, anti-apoptosis, involvement in protein folding) or subcellular localization (e.g., cytoplasm, endoplasmic reticulum, mitochondria, nucleus), providing valuable insights into their specific roles [10].

The chaperone function is the primary and most essential role of HSPs, often accomplished through the synergistic actions of different subfamily members. For example, HSP40 recognizes and binds unfolded client proteins, subsequently recruiting HSP70 to initiate the ATP-dependent folding process. Through their chaperone function, HSPs are vital for maintaining intracellular homeostasis and resisting external stress, primarily by protecting nascent polypeptide chains and facilitating proper protein folding, transport, and degradation [11, 12].

In addition to their chaperone function, HSPs maintain intracellular homeostasis by precisely regulating apoptosis, exhibiting both anti-apoptotic and pro-apoptotic roles depending on the context. Regarding their anti-apoptotic function, HSPs primarily act by interfering with apoptotic signaling pathways and preserving the integrity of cellular structures [13–16]. Conversely, during immune effector cells attack on target cells, certain HSPs can cooperate to promote apoptosis [15]. Beyond maintaining intracellular balance, HSPs also possess important immunomodulatory functions and can serve as key immunogens in immune responses against pathogens [17]. Their immunomodulatory role operates at two levels: the release of HSPs into the extracellular space can regulate innate immunity, while their involvement in antigen presentation can induce acquired, specific immunity [18, 19]. In summary, through their chaperone function, HSPs play an indispensable role in maintaining cellular homeostasis and regulating immune responses.

The giant salamanders (*Andrias* spp.) belong to the order Caudata and the family Cryptobranchidae and are the largest extant amphibian species. As an ancient relict lineage, often referred to as “living fossils” their evolutionary history dates back approximately 160 million years, making them an ideal model for studying biological evolution and ecological adaptation [20]. The Chinese giant salamander, *Andrias davidianus* (Blanchard, 1871), was once widely distributed across 17 Chinese provinces within the Yangtze, Yellow, and Pearl River basins. However, since the 1950s, habitat destruction and overhunting have caused a sharp decline in its wild population, and its habitats have become increasingly isolated and fragmented [21, 22]. Due to its endangered status, the International Union for Conservation of Nature (IUCN) has classified *A. davidianus* as Critically Endangered (CR), and its wild population is also listed as a National Class II Key Protected Wild Animal in China [20]. As an ancient amphibian, *A. davidianus* occupies a unique ecological niche, with its survival closely linked to the aquatic environment. It is highly sensitive to changes in water quality, particularly water temperature. The species thrives within a temperature range of 15–25 °C; when the temperature exceeds this range, stress responses such as reduced appetite, sluggish activity, and states resembling aestivation or hibernation may occur [23, 24].

Like most aquatic organisms, *A. davidianus* exhibits strong stress responses and significant physiological changes when exposed to sudden environmental fluctuations [25]. One of the most notable phenotypes is the massive secretion of white mucus from the skin mucous glands upon stimulation, which helps the salamander evade predators and promotes wound healing [26]. Early research on temperature stress in *Andrias* primarily focused on behavioral responses. For example, studies found that high temperatures inhibit the feeding activity of *A. davidianus*, with feeding frequency decreasing as temperature rises, potentially leading to mortality [27]. Subsequent studies expanded to physiological and biochemical levels, revealing that temperature stress significantly increases lactate dehydrogenase (LDH) activity and malondialdehyde (MDA) content in the liver, indicating cellular damage and oxidative stress. Histological analysis has further demonstrated that high-temperature stress induces hepatocyte abnormalities, fat accumulation, and ultrastructure alterations, including changes to the endoplasmic reticulum and nucleus [28]. More recently, research has shifted to the molecular level. For example, studies have shown that both high- and low-temperature stress upregulate the expression of *CgHsp901* and *CgHsp902* genes in the skin, muscle, and heart tissues of *A. davidianus*. However, in the intestine, pancreas, and stomach, these genes are upregulated under high-temperature conditions but downregulated under low-temperature conditions, indicating clear tissue specificity and temperature-dependent response patterns. This highlights their critical roles in the salamander’s adaptation to extreme temperatures [29]. Despite these advances, molecular research on temperature stress in *A. davidianus* remains limited, primarily focusing on a few HSP genes, which restricts our understanding of the molecular regulatory networks involved in the salamander’s response to temperature and other environmental stresses.

Based on multi-tissue transcriptome data, this study systematically identified members of the HSP gene family in *A. davidianus* and analyzed their sequence characteristics and phylogenetic relationships. It further investigated changes in their expression patterns under combined stress from temperature fluctuations and pathogen infection. The research aims to comprehensively elucidate the stress response mechanisms of HSPs in *A. davidianus*, providing new insights into its environmental adaptive evolution and offering a critical theoretical foundation for developing conservation strategies for wild populations and artificial breeding, such as managing high-temperature conditions and disease outbreaks.

## Materials and methods

### Data source and experimental design

The transcriptomic data were obtained from a previous study conducted by our research group (unpublished Master’s thesis [30]), which generated gene expression profiles from artificially farmed *A. davidianus* infected with *Aeromonas hydrophila* (Chester, 1901) Stanier, 1943 under both normal and elevated temperature conditions. The experimental design included three treatments: (I) Blank control (T0): uninfected healthy individuals, serving as the baseline control; (II) Infection at normal temperature: conducted at a winter indoor water temperature of approximately 10 °C, with four post-infection time points (T1: 12 h, T2: 36 h, T3: 60 h, T4: 15 d); and (III) Infection at elevated temperature: conducted at a controlled water temperature of 25 ± 0.5 °C, with four parallel time points (T5: 12 h, T6: 36 h, T7: 60 h, T8: 15 d).

The salamanders used in this study were purchased from a local commercial farm that legally certified for the artificial rearing and breeding of captive-bred *A. davidianus* of the second and subsequent generations. All individuals were of the same age (3 years) and reared under identical conditions, with an average body weight of approximately 230 g. Salamanders assigned to the experimental groups were challenged via intraperitoneal injection with 0.1 mL of an *A. hydrophila* suspension, while those in the control group received an equal volume of sterile physiological saline. Prior to sample collection, all salamanders were euthanized using MS-222 (Macklin, Shanghai, China) at a concentration of 80 mg/kg. Kidney, liver, and spleen tissue samples were collected from the salamanders in each treatment group. Each treatment group included three biological replicates, resulting in a total of 81 samples (9 treatment groups × 3 tissue types × 3 replicates).

### RNA extraction, sequencing, assembly and annotation

Total RNA was extracted from all samples using the RNAprep Pure Animal Tissue Total RNA Extraction Kit (DP431; TIANGEN, Beijing, China). Subsequently, eukaryotic mRNA was enriched using magnetic beads with Oligo(dT) and randomly fragmented into short segments by ultrasonication. Using the fragmented mRNA as a template, the first strand of cDNA was synthesized via reverse transcription, followed by synthesis of the second strand using DNA polymerase I. The purified double-stranded cDNA was end-repaired, A-tailed, and ligated with sequencing adapters. Fragments approximately 200 bp in length were selected using AMPure XP beads for PCR amplification, ultimately constructing the sequencing libraries. Library concentration was accurately quantified using Qubit 2.0, and RNA integrity was assessed with the Agilent 2100 system. Paired-end sequencing (PE150) was performed on the Illumina sequencing platform.

Raw sequencing reads were initially assessed for quality using FastQC (v0.11.2) [31]. Subsequently, Trimmomatic (v0.39) [32] was employed to filter the raw data by removing low-quality reads, adapter sequences, and reads with an N base ratio exceeding 10%, resulting in high-quality clean reads. *De novo* transcriptome assembly was performed using Trinity software [33] with default parameters. The completeness of the assembled transcript set was evaluated using BUSCO (Benchmarking Universal Single-Copy Orthologs) software [34]. To reduce redundancy caused by alternative splicing (isoforms), the longest transcript per locus was selected as a unigene based on the Trinity assembly. Further clustering and redundancy reduction were conducted using CD-HIT-EST (with a similarity threshold of 0.95) to minimize false positives in subsequent expression level analyses. Using Trinity’s built-in tool align_and_estimate_abundance.pl, clean reads were aligned back to the assembled unigene reference sequences to obtain gene counts, which were then converted to FPKM (Fragments Per Kilobase of transcript per Million mapped reads) values to quantify gene expression levels.

TransDecoder software was used to predict coding sequences (CDS) and amino acid sequences. Sequences were rigorously filtered based on completeness labels (complete, 5prime_partial, 3prime_partial) generated by TransDecoder. Functional annotation was performed by conducting BLAST alignments (E-value < 1e-5) of the predicted sequences against the NR, SwissProt, and COG/KOG databases to ensure comprehensive coverage of key domains. The raw transcriptome sequencing data generated in this study have been deposited in the NCBI Sequence Read Archive (SRA) database. Data were uploaded using the FileZilla (v3.39.2) FTP client, and the corresponding BioProject accession number is PRJNA1394661.

### Identification of HSPs in *A. davidianus*

The identification of HSP genes followed this procedure: Known HSPs sequences from *Xenopus tropicalis* (Gray, 1864) and *Microcaecilia unicolor* (Duméril & Bibron, 1841), along with previously reported sequences of *A. davidianus* [35, 29], were used as query sequences for a BLASTP alignment against the longest transcripts obtained from the salamander transcriptome assembly. An E-value threshold of 1 × 10⁻⁵ was set to control for false positives. Additionally, conserved domains of HSP families were retrieved from the InterPro database (https://www.ebi.ac.uk/interpro/) (HSP20: PF00011, HSP40: PF00226, HSP60: PF00118, HSP70: PF00012, HSP90: PF00183), and hidden Markov models (HMMs) were constructed for a secondary screening of the BLAST results using the HMMER software package [36], with an E-value threshold of 1 × 10⁻³. The intersection of the results from both methods was taken as the set of potential HSP genes in *A. davidianus*. Redundant sequences were removed, and the Pfam or InterPro databases were used to verify the presence of the corresponding conserved HSP domains, ultimately yielding the non-redundant members of the HSP gene family of *A. davidianus*. Furthermore, to support the identification results, an orthologous analysis was performed on the protein sequence sets of *A. davidianus*, *X. tropicalis*, and *M. unicolor* using OrthoFinder (v2.5.4) [37] Sequence alignment was conducted with DIAMOND (v2.0.15) (E-value ≤ 1 × 10⁻⁵) [38], and all identified HSPs were assigned to orthologous groups using a graph clustering algorithm.

### Sequence analysis and phylogenetic tree construction

To investigate the physicochemical properties of the HSP family members in *A. davidianus***—**including molecular weight, isoelectric point, and hydrophobicity**—**we used the Expasy ProtParam online tool (https://web.expasy.org/protparam/) to analyze their theoretical isoelectric point (pI), molecular weight, instability index, and aliphatic index. These parameters provide valuable insights into protein structure, function, and stability. To elucidate the evolutionary relationships among the HSP families of *A. davidianus*, *X. tropicalis* and *M. unicolor*, we performed multiple sequence alignment of the HSP sequences from these three species using the L-INS-i algorithm in MAFFT software [39]. Based on the alignment results, a phylogenetic tree was constructed using the maximum likelihood method in IQ-TREE 2 [40]. Bootstrap support (BS) values for the phylogenetic tree were calculated using the ultrafast bootstrap method with 1000 replicates and were further evaluated by SH-aLRT values, as recommended in the IQ-TREE manual. Model selection followed the Bayesian Information Criterion (BIC), with the optimal substitution model (JTT+R5) determined using ModelFinder [41]. The tree was visualized using the EvolView online platform (http://www.evolgenius.info/evolview/#/treeview). Finally, based on the clustering results, the HSP sequences from *A. davidianus*, *X. tropicalis* and *M. unicolor* were identified and classified into gene subfamilies.

### Analysis of gene structure characteristics

To gain an in-depth understanding of the structural characteristics of the HSP genes in *A. davidianus*, we conducted an analysis of their coding sequences (CDS), untranslated regions (UTRs), conserved motifs, and domains. First, to identify conserved motifs, the HSP sequences of *A. davidianus* were submitted to the MEME online tool (https://meme-suite.org/meme/tools/meme), with parameters set to “Classic” mode and “Zero or One Occurrence per Sequence” to predict 15 motifs [42]. Second, the known conserved domains of the HSP family members in *A. davidianus* were retrieved from the NCBI database (https://www.ncbi.nlm.nih.gov/Structure/bwrpsb/bwrpsb.cgi), and their annotation information was extracted [43]. Finally, the EvolView online platform was used for integrated visualization of gene structures, conserved motifs, and domains.

### Protein function prediction

To predict the functional characteristics of the HSP family members in *A. davidianus*, we employed a series of bioinformatics methods using online software and databases. First, the TMHMM Server v.2.0 (https://services.healthtech.dtu.dk/services/TMHMM-2.0/) was used to predict protein transmembrane domains, and SignalP 6.0 (https://services.healthtech.dtu.dk/services/SignalP-6.0/) was used to analyze the presence and location of signal peptides. Simultaneously, the NetNGlyc 1.0 Server (https://services.healthtech.dtu.dk/services/NetNGlyc-1.0/) was employed to predict N-linked glycosylation sites. Second, the WoLF PSORT (https://wolfpsort.hgc.jp/) and SOPMA (https://npsa-lyon.ibcpc.fr/cgi-bin/npsa_automat.pl?page=/NPSA/npsa_sopma.html) online tools were utilized to predict the subcellular localization [42] and secondary structure of the HSP family members, respectively. Third, the Phyre2 database (http://www.sbg.bio.ic.ac.uk/~phyre2/) was used to model the three-dimensional structures of the target proteins. Finally, to enhance the reliability of the prediction models and their representativeness of actual structures [44], we excluded prediction results with both confidence and coverage below 75%.

### Protein-protein interaction network

To construct the interaction network of the HSP genes in *A. davidianus*, we used the STRING database (https://string-db.org/cgi/) to predict interaction relationships. The HSP sequences of *A. davidianus* were orthologously mapped to *X. tropicalis*, which was then used as the query input for the STRING database to obtain interaction information. Subsequently, Cytoscape (v3.6.1) [45] was employed to visualize the interaction data output by STRING and to analyze the network’s topology properties [46, 47].

### Differential expression analysis

To investigate the expression patterns of *AndHSP* genes under temperature and pathogen stress, differential expression analyses were performed in the liver, spleen, and kidney using previously established transcriptomic datasets. Samples were assigned to a single factor “group”, representing the full experimental condition: Control (T0), Normal‑temperature infection (T1–T4), and Elevated‑temperature infection (T5–T8). Differential expression analysis was conducted using DESeq2 [48] with the model design: design = ~ group. This design was selected because the absence of an uninfected high‑temperature control precluded the estimation of a formal Temperature × Infection interaction term. Instead, the biological effect of temperature on infection‑induced transcriptional responses was assessed through targeted contrasts. Three categories of comparisons were performed using the results() function with explicitly defined contrasts (contrast = c(“group”, “A”, “B”)): (1) Infection effect: each infected group versus the control (T0); (2) Temporal effect: pairwise comparisons among infection time points within the same temperature condition; and (3) Temperature modulation: comparisons between matched time points under normal‑ and elevated‑temperature infections (T1 vs. T5, T2 vs. T6, T3 vs. T7, and T4 vs. T8). These contrasts capture the functional equivalent of a Temperature × Infection interaction within the infected conditions.

Each comparison was performed independently to identify genes that were differentially expressed across the three aforementioned categories. Firstly, the raw count values and FPKM values for all identified HSP genes in *A. davidianus* were extracted from the transcriptome expression annotation files. Next, DESeq2 was employed to analyze the raw count data, calculating the log₂FoldChange and adjusted P-value (P-adjust) for each gene. HSP genes with |log₂FoldChange| > 1 and P-adjust < 0.05 were considered differentially expressed genes (DEGs) [49]. Finally, to visually represent the expression patterns, the FPKM values of the HSP genes were log₂-transformed, and a heatmap was generated using the BIC online platform (https://www.bic.ac.cn/BIC/#/).

### Quantitative real-time PCR validation

To verify the reliability of the transcriptome sequencing results, we selected nine differentially expressed HSP genes of *A. davidianus*, identified through transcriptome analysis, for validation by real-time quantitative PCR (qPCR) using the same batch of preserved liver samples. Specific primers for these nine target genes were designed using Primer 5 (Table 1), along with primers for the β-actin gene, which served as the reference gene for normalization in this study. Total RNA was extracted from the samples using the RNA Easy Fast Tissue/Cell Kit (DP451, Tiangen Biotech, Beijing). cDNA was synthesized following the instructions provided with the FastSensi gDNA Dispelling RT SuperMix (KR138, Tiangen Biotech, Beijing). The qPCR reactions were performed on an Applied Biosystems StepOnePlus real-time PCR system (Thermo Fisher Scientific, USA), with three experimental replicates for each sample.

**Table 1:** Primer sequences for reference and target genes in qPCR validation

Primer name	Primer sequence
*β-actin* F	CCATCAATCGTCCACCGTA
*β-actin* R	CGCATCAAGCACCAGAAAC
*AndHSP40-17* F	GAAGAATGGGAGCAGAAGA
*AndHSP40-17* R	GATTAGGATGGAGCGATGT
*AndHSP70-02* F	AGCACGCTTTGAGGAACTG
*AndHSP70-02* R	CTTTGTCCATCTTGGCATC
*AndHSP70-10* F	CTCCTCTGCCACTCTTGTC
*AndHSP70-10* R	TCACTGTGCCTGCATACTT
*AndHSP70-15* F	TATCCGTGAAGGCAACATA
*AndHSP70-15* R	TCTAAAGGACCATCAGTCG
*AndHSP70-17* F	GTCCTCATCAAACGCAACT
*AndHSP70-17* R	GCTCTTTCACCCTCATACAC
*AndHSP70-18* F	TGACTCACATTTGGGAACA
*AndHSP70-18* R	GATTGCATGAATGATAGCG
*AndHSP70-20* F	GCGGTCGCTGAACTCGTTG
*AndHSP70-20* R	ATGTCGGTGGTGGGCTTTG
*AndHSP90-02* F	TGTTGGATCTGGCAGTGGT
*AndHSP90-02* R	TGGCTCCTCCTCAACCTTT
*AndHSP90-03* F	GGAGAAGGAGCGTAACAAG
*AndHSP90-03* R	CCTCATCAGAGCCAACATC

The qPCR reaction system had a total volume of 20 µL, containing 2 µL of template cDNA, 0.6 µL each of forward and reverse primers (10 µM), 10 µL of 2× FastReal qPCR PreMix (SYBR Green), 2 µL of 50× ROX Reference Dye, and 4.8 µL of RNase-free ddH₂O. The amplification program was set as follows: pre-denaturation at 95 °C for 2 min, followed by 40 cycles of denaturation at 95 °C for 5 s and annealing/extension at 56 °C for 15 s. Relative gene expression levels were calculated using the 2 ^(−ΔΔCt)^ method [50], and results are presented as mean ± standard error of the mean (mean ± SEM). One-way analysis of variance (ANOVA) was performed using GraphPad Prism 10.1.2 software, with statistical significance defined as *p < 0.05, **p < 0.01, ***p < 0.001, and ****p < 0.0001.

### Weighted gene correlation network analysis

To investigate gene co-expression patterns, weighted gene co-expression network analysis (WGCNA) was performed using R (v4.3.3) and the WGCNA package (v1.69) [51]. Three sample groups were strategically selected to capture the early stress response: the control (T0), and the earliest infection time points under normal (T1) and elevated (T5) temperature conditions. This selection was based on the biological rationale that HSPs are typically induced during the initial onset of stress. By focusing on these early stages, the network analysis aimed to resolve the primary transcriptional modules activated by pathogen challenge and temperature elevation, thereby minimizing confounding effects arising from long-term acclimation or recovery processes at later time points (T2–T4, T6–T8).

Firstly, the pickSoftThreshold function was used to calculate and determine a soft threshold of 28, ensuring that the constructed network satisfied the scale-free topology criteria. Building upon this and following previous studies [52], hierarchical clustering was performed using the hclust function to generate a gene clustering dendrogram, which was then used to partition gene modules. The gene sets of each module were subsequently extracted for further analysis.

To quantify the strength of association between each module and the examined groups, the first principal component of the gene expression levels within a module was defined as the Module Eigengene (ME). The Bicor algorithm was used to calculate the correlation between each ME and the three examined groups. Following a previous study [53], the overall strength of association between modules and examined groups was assessed by calculating the Module Significance (MS). Based on a comprehensive evaluation of both MS and ME correlations, the key modules most strongly associated with the two treatment groups were selected. Members of the HSP gene family were then extracted from these key modules for further analysis.

## Results

### Identification and classification of HSP genes in *A. davidianus*

The BUSCO assessment of transcriptome assembly completeness indicated a completeness level of 84.5%, reflecting a high-quality assembly. A total of 1,239,061 unigenes were obtained, with an average length of 479 bp and an N50 length of 918 bp. Through BLAST homology alignment and HMMER conserved domain searches, followed by stringent screening, we identified 72 HSP genes from the transcriptome of *A. davidianus*. These genes were designated as the HSP gene family of *A. davidianus* (*AndHSP*). Based on subfamily classification and increasing molecular weight, the genes were grouped into five subfamilies: *AndHSP20* (9 members), *AndHSP40* (29 members), *AndHSP60* (7 members), *AndHSP70* (21 members), and *AndHSP90* (6 members) (Supplementary Table 1). Orthologous group analysis using OrthoFinder revealed that all candidate HSP genes identified in *A. davidianus* clustered within the same orthogroup as at least one known HSP member from the reference species. Additionally, genes from different subfamilies formed distinct orthogroups or well-defined subgroups. These results effectively validate the accuracy of the *AndHSP* gene identification (Supplementary Table 2).

### Analysis of the physicochemical properties of *AndHSPs*

To systematically analyze the physicochemical properties of each subfamily of the *AndHSPs*, we examined their amino acid sequence length, theoretical isoelectric point (pI), instability index (II), and hydrophilicity/hydrophobicity. The results are summarized in Supplementary Table 3. The main findings are as follows:

1. *AndHSP20* subfamily: The nine members range in length from 143 amino acids (*AndHSP20-05*) to 285 amino acids (*AndHSP20-07*). This subfamily predominantly encodes acidic proteins (pI < 7); most members are predicted to be unstable (II ≥ 40), and all are hydrophilic.
2. *AndHSP40* subfamily: The 29 members vary widely in length, ranging from 149 amino acids (*AndHSP40-16*) to 2,240 amino acids (*AndHSP40-11*). Among them, nine encode acidic proteins, while twenty encode basic proteins (pI > 7). Nine members are predicted to be stable (II < 40), and twenty are unstable; all members are hydrophilic.
3. *AndHSP60* subfamily: The seven members range in length from 115 amino acids (*AndHSP60-07*) to 576 amino acids (*AndHSP60-06*). Five members encode acidic proteins, while two encode basic proteins. All members are predicted to be stable; five are hydrophilic and two (*AndHSP60-05* and *AndHSP60-07*) exhibit hydrophobic characteristics.
4. *AndHSP70* subfamily: The 21 members range in length from 100 amino acids (*AndHSP70-13*) to 983 amino acids (*AndHSP70-06*). Fifteen members encode acidic proteins, five encode basic proteins, and one encodes a neutral protein. Twelve members are stable, while nine are unstable; all members are hydrophilic.
5. *AndHSP90* subfamily: The six members range in length from 103 amino acids (*AndHSP90-04*) to 327 amino acids (*AndHSP90-03*). Five members encode acidic proteins, while one encodes a basic protein. All members are predicted to be stable and hydrophilic.

### Phylogenetic analysis

**Fig. 1:**
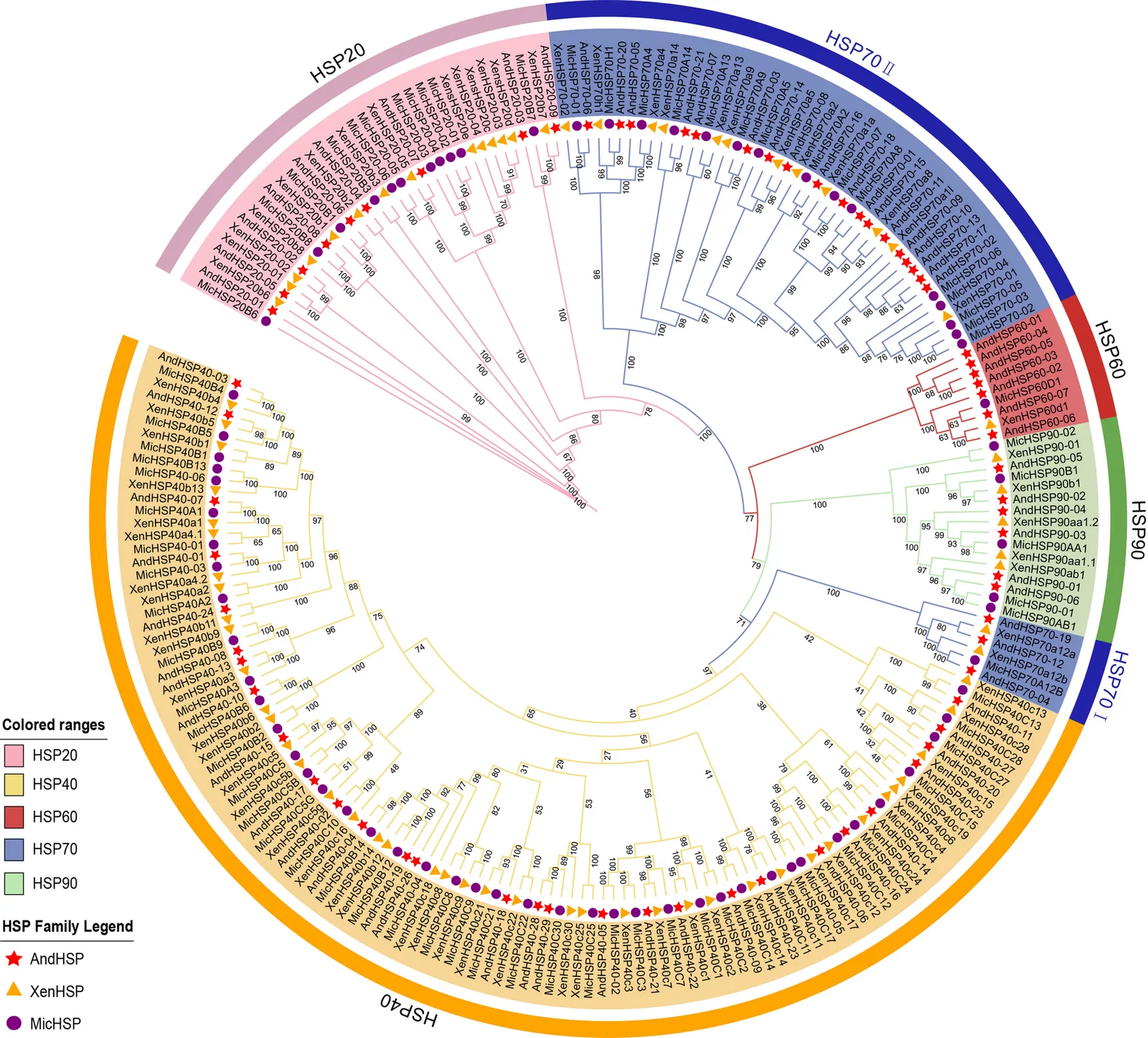
Phylogenetic relationships of heat shock proteins from *Andrias davidianus*, *Xenopus tropicalis*, and *Microcaecilia unicolor*

To investigate the evolutionary relationships of the *AndHSPs*, a phylogenetic tree was constructed using HSP sequences from *A. davidianus*, *X. tropicalis* and *M. unicolor* (Fig. 1, SH-aLRT values are provided in Supplementary Fig. 1). The results reveal that HSPs generally form distinct and well-supported evolutionary clades corresponding to their respective gene subfamilies, indicating strong evolutionary conservation within each subfamily. Members of the HSP70 subfamily cluster into two distinct branches: one branch (HSP70-I) contains fewer genes and clusters as a sister group to the HSP40 subfamily (BS = 71), then clusters with the HSP90 subfamily (BS = 79), followed by clustering with HSP60 (BS = 77); the other branch (HSP70-II), however, shows a sister group relationship with the aforementioned subclade [(((HSP70-I, HSP40), HSP90), HSP60)] and contains the majority of the genes in the HSP70 subfamily.

Gene family expansion analysis revealed species-specific differences. The HSP40 subfamily had the highest number of genes across all three species, but its distribution varied significantly. For instance, *M. unicolor* (*n* = 42) has approximately 1.5 times more HSP40 genes than *A. davidianus* (*n* = 29). In contrast, the HSP60 subfamily underwent significant expansion in *A. davidianus* (*n* = 7), forming a species-specific evolutionary cluster, whereas *X. tropicalis* and *M. unicolor* each possess only a single HSP60 gene. The number of genes in the HSP90 subfamily was relatively similar among the three species (*A. davidianus*, *n* = 6; *X. tropicalis* and *M. unicolor*, *n* = 5), with orthologous genes clustering together, indicating that this subfamily is the most evolutionarily conserved. In summary, phylogenetic analysis shows that while HSP gene families in amphibians exhibit overall evolutionary conservation, different subfamilies display diverse expansion patterns and species-specific evolutionary traits.

### Analysis of gene structure characteristics

**Fig. 2:**
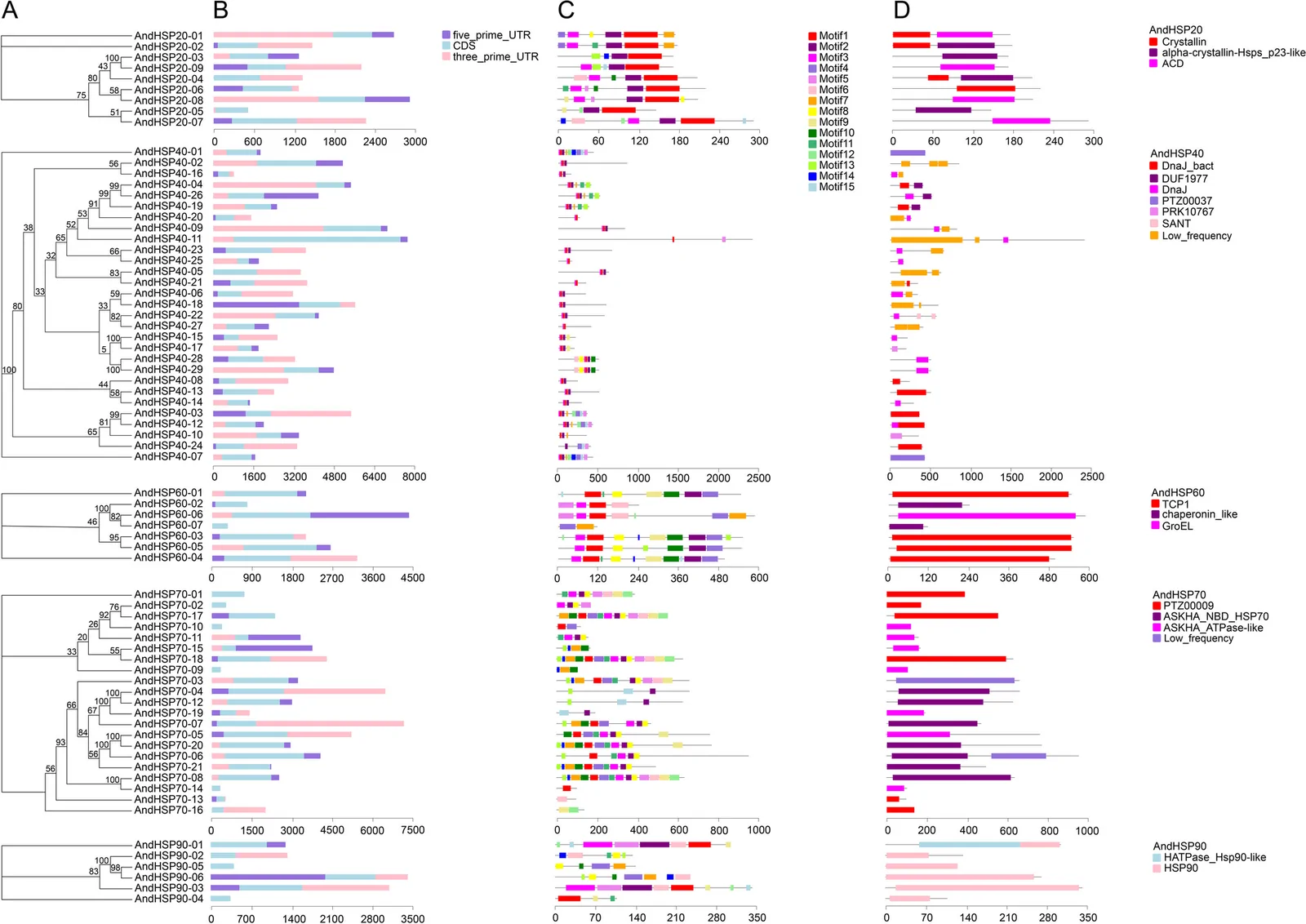
Phylogenetic tree (**A**), gene structure (**B**), conserved motifs (**C**), and domain architecture (**D**) of the five heat shock protein subfamilies in *Andrias davidianus*

To gain a deeper understanding of the structural characteristics of the HSP subfamilies in *A. davidianus*, we constructed phylogenetic trees for the five subfamilies based on their evolutionary relationships (Fig. 2A) and comprehensively compared their gene structures, conserved motifs, and domain compositions (Fig. 2B-D). The CDS lengths of genes from different subfamilies varied significantly, ranging from the shortest at 302 bp in the *AndHSP70* subfamily to the longest at 6722 bp in the *AndHSP40* subfamily. Most members contained typical UTRs, with exceptions such as *AndHSP60-07*, four members of the *AndHSP70* subfamily, and *AndHSP90-04* and *AndHSP90-05*, which lacked typical UTRs (Fig. 2B). Regarding conserved motifs, 15 motifs were identified using the MEME Suite. The number and length of motifs varied among subfamilies; the *AndHSP70* subfamily exhibited the highest number of motifs (ranging from 2 to 14), while the *AndHSP20* subfamily showed the greatest variation in motif length (ranging from 7 to 50 amino acids). Notably, members clustering closely on the phylogenetic tree generally shared similar motif compositions. Overall, the *AndHSP20*, *AndHSP60*, and *AndHSP70* subfamilies showed the highest motif conservation (Fig. 2C).

Protein domain analysis revealed the specificity and internal diversity of each subfamily. (1) *AndHSP20*: All members contain the signature alpha-crystallin domain (ACD), which corresponds to the first and second conserved motifs. (2) *AndHSP40*: This subfamily exhibits the most diverse domain composition, primarily including the DnaJ and its bacterial homolog (DnaJ_bact) domains, with conserved motifs 1–3 overlapping with the DnaJ domain. Individual members, such as *AndHSP40-27*, also contain unique domains like PLN03085. (3) *AndHSP60* and *AndHSP90*: Members of both subfamilies contain their respective subfamily-specific domains, showing high conservation. *AndHSP90-01* forms an independent branch on the phylogenetic tree due to an additional HATPase_Hsp90-like domain at its N-terminus. (4) *AndHSP70*: This subfamily shows domain diversity, including ASKHA_ATPase-like and PTZ00009 domains. *AndHSP70-06* contains four different types of domains (Fig. 2D).

### Protein function prediction

To comprehensively analyze the potential functions of *AndHSPs*, we predicted their subcellular localization, transmembrane domains, glycosylation sites, and secondary and tertiary structures (Supplementary Table 3, Supplementary Figs. 2, 3, 4). The main findings are summarized as follows:

1. Subcellular localization and transmembrane characteristics: Among the 72 identified *AndHSPs*, most (40 members) were predicted to localize in the cytoplasm. The localization of the remaining members was predicted as follows: nucleus (*n* = 14), mitochondria (*n* = 9), extracellular space (*n* = 8), plasma membrane (*n* = 8), endoplasmic reticulum (*n* = 4), and cytoskeleton (*n* = 1). Transmembrane domain predictions implied that all *AndHSP20*, *AndHSP60*, and *AndHSP90* subfamily members lacked transmembrane helices, indicating that they are localized within the plasma membrane. In contrast, 7 members of the *AndHSP40* subfamily were predicted to contain transmembrane domains. Members of the *AndHSP70* subfamily displayed diversity in the positions of their signal peptides: 3 had an N-terminal signal peptide, 13 were located in the plasma membrane, and 8 were found outside the plasma membrane.
2. Glycosylation sites: The number of N-linked glycosylation sites varied significantly across subfamilies. All *AndHSP20* members contained 1-2 sites. Other subfamilies contained both glycosylated and non-glycosylated members, with the *AndHSP40* subfamily having the highest number of glycosylation sites (up to 9). However, this subfamily also had the most members without glycosylation sites (*n*=11).
3. Protein structure: Secondary structure predictions showed that random coils and alpha-helices were the main structural elements in all *AndHSPs*, but their relative proportions varied among subfamilies. For example, random coils predominated in *AndHSP20* and *AndHSP40*, while alpha-helices were more prominent in *AndHSP60*, *AndHSP70*, and *AndHSP90*. Homology modeling based on Phyre2 indicated that the three-dimensional structures of members within the same subfamily were generally similar, with some variation, particularly in the large *AndHSP70* subfamily. This suggests potential functional differentiation (Supplementary Fig. 4).

### Protein-protein interactions

To predict functional associations, a protein-protein interaction (PPI) network for *AndHSPs* was constructed based on known interaction models from *X. tropicalis* (Supplementary Fig. 5). The analysis revealed that all *AndHSPs* could map to their orthologous proteins in *X. tropicalis*, with the *AndHSP70* subfamily showing the highest number of homologous members (8) corresponding to the single hspa1l gene of *X. tropicalis*. In the predicted PPI network, a total of 56 protein nodes and 745 interaction edges were included. Using a threshold of the top 9% of Degree values, five hub proteins were identified from the PPI network. Among them, *AndHSP70-03* exhibited the highest degree value (Degree = 47), indicating the largest number of direct interacting partners. In contrast, the interaction relationships of some members were weaker. For instance, *AndHSP20-07* was not predicted to have any interaction connections, and *AndHSP40-06* also displayed a relatively low degree value (Degree = 3).

### Gene expression analysis

**Fig. 3:**
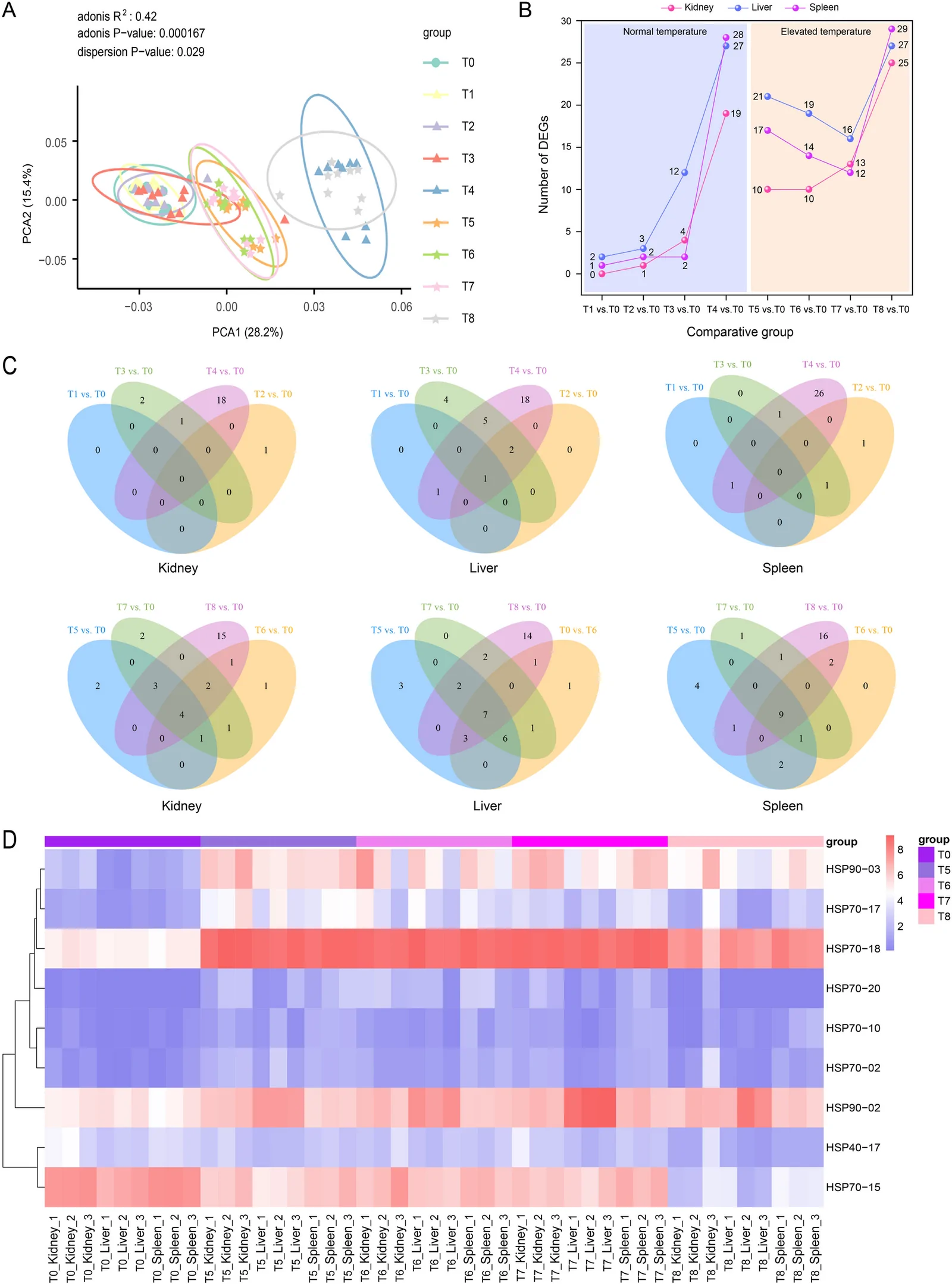
Differential expression analysis of heat shock protein genes in the *Andrias davidianus* under normal and elevated temperature infection. Notes: (**A**) Principal component analysis (PCA) shows sample clustering, where circles represent the control group, triangles represent the normal temperature infection group, and stars represent the elevated temperature infection group. (**B**) Bar plot of the number of DEGs under two infection conditions. (**C**) Venn diagram of shared DEGs across three tissues. (**D**) Heatmap of nine DEGs co-expressed across multiple tissues under the elevated temperature infection conditions

To analyze the expression dynamics of *AndHSPs* genes under the stress of temperature and pathogen infection, we conducted a multi-level expression analysis. The PCA revealed clear separation between the samples from the normal temperature infection group, the elevated temperature infection group, and the control group, indicating that both treatments induced significant transcriptomic responses (Fig. 3A). Differential expression analysis between groups identified a series of *HSP* genes, the expression of which displayed distinct temporal patterns (Supplementary Table 4). Under both normal and elevated temperature infection treatments, the number of DEGs increased with the duration of infection, though the patterns differed: in the normal temperature group, the number of DEGs gradually increased, whereas in the elevated temperature group, the DEGs peaked early in the infection (T5) and remained elevated (Fig. 3B).

We also identified genes exhibiting sustained responses across specific tissues. Under normal temperature infection conditions, only one gene (*AndHSP70-20*) in the liver was differentially expressed at all time points. In contrast, elevated temperature infection triggered a broader and more sustained tissue response, with 4, 7, and 9 *AndHSPs* genes showing differential expression at all time points in the kidney, liver, and spleen, respectively (Fig. 3C). Among the nine genes that were consistently differentially expressed across tissues under elevated temperature infection, the majority (*n* = 6) were from the *AndHSP70* subfamily, followed by *AndHSP90* (*n* = 2) and *AndHSP40* (*n* = 1). The expression patterns of these nine core genes showed that most were upregulated (*n* = 7), while *AndHSP40-17* and *AndHSP70-15* exhibited downregulation trends (Fig. 3D). Lastly, an heatmap analysis of all *AndHSPs* gene expression levels (Supplementary Fig. 6) revealed that some genes exhibited stable expression across treatments and tissues. In contrast, compared with members of the *AndHSP20* and *AndHSP40* subfamilies, genes in the *AndHSP60*, *AndHSP70*, and *AndHSP90* subfamilies, showed more significant changes in expression over time (from T1 to T4, and from T5 to T8), primarily upregulation relative to T0 (Supplementary Fig. 6). Additionally, expression exhibited tissue specificity, with five *AndHSP60* genes showing significantly higher expression levels in the spleen compared to the kidney and liver (Supplementary Fig. 6).

### qPCR expression analysis and validation

**Fig. 4:**
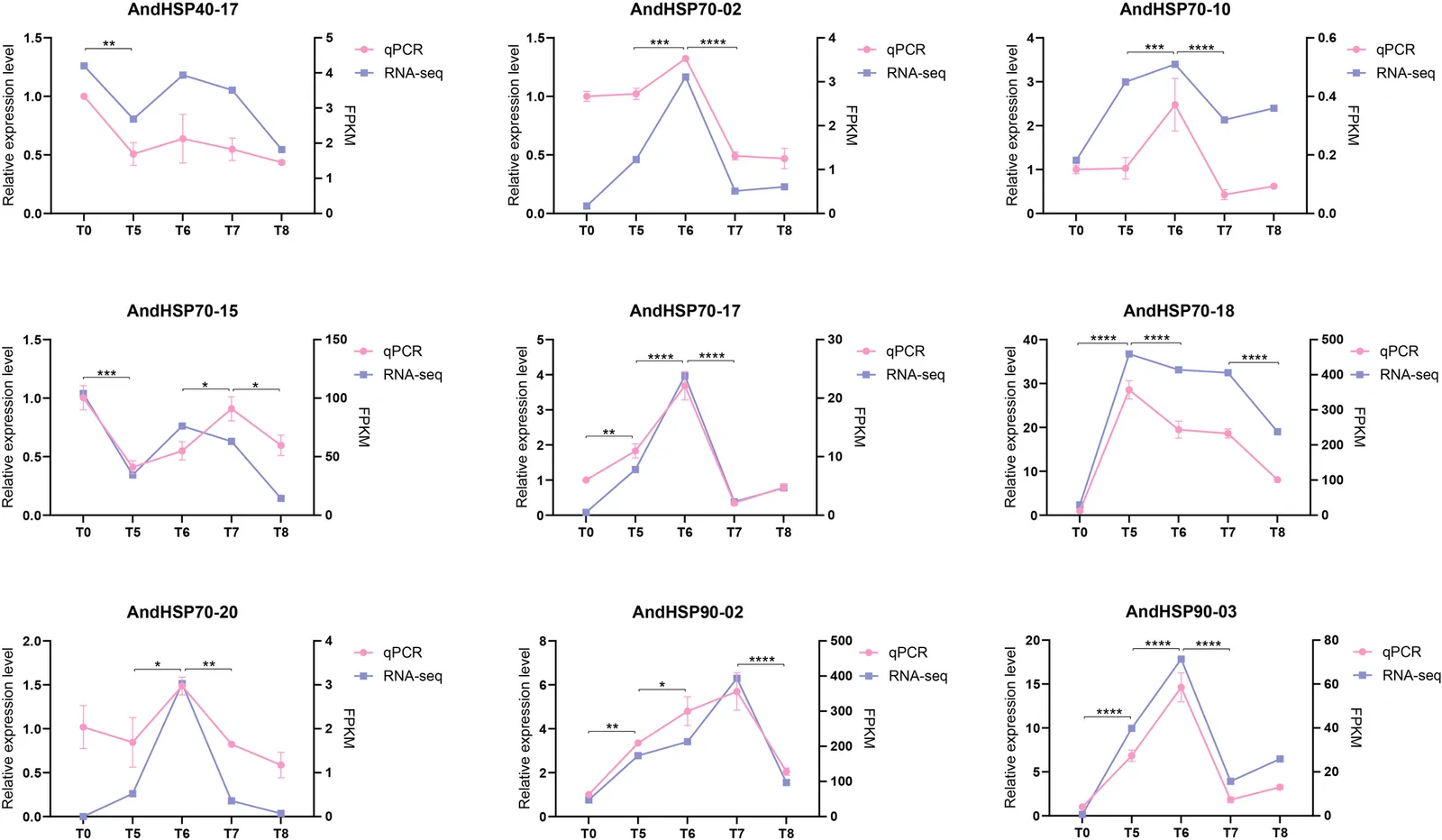
Validation by qPCR of nine differentially expressed heat shock protein genes under elevated temperature infection. Notes: Comparison between qPCR relative expression levels (left Y-axis) and RNA-Seq FPKM values (right Y-axis)

To verify the reliability of the transcriptome sequencing results, nine *AndHSPs* genes (*AndHSP40-17*, *AndHSP70-02*, *-10*, *-15*, *-17*, *-18*, *-20*, *AndHSP90-02*, *-03*) that exhibited the most significant differential expression under the elevated temperature infection treatment were selected for real-time qPCR validation. The qPCR results showed good overall agreement with the RNA-Seq data. Among the nine validated genes, the expression trends (upregulation or downregulation) of seven genes were completely consistent with the RNA-Seq results (Fig. 4). However, the expression patterns of the remaining two genes, *AndHSP70-15* and *AndHSP70-20*, showed slight discrepancies at specific time points. For instance, RNA-Seq indicated that the expression of *AndHSP70-15* first increased and then decreased during the T5-T7 period, while qPCR results showed a continuous increase. Similarly, RNA-Seq suggested that *AndHSP70-20* was upregulated during the early infection stage (T0-T5), whereas qPCR showed a downregulation trend. Despite these minor discrepancies, the expression patterns of the majority of genes were highly consistent, supporting the reliability of the transcriptomic data in this study. Further analysis of the dynamic expression patterns of these nine key genes revealed that the expression levels of most peaked during the early to middle stages of infection (T5-T7), before decreasing at the T8 time point. Notably, *AndHSP70-18* showed a sharp upregulation early in the infection (T5), exhibiting a characteristic of highly inducible expression.

### Weighted gene correlation network analysis

**Fig. 5:**
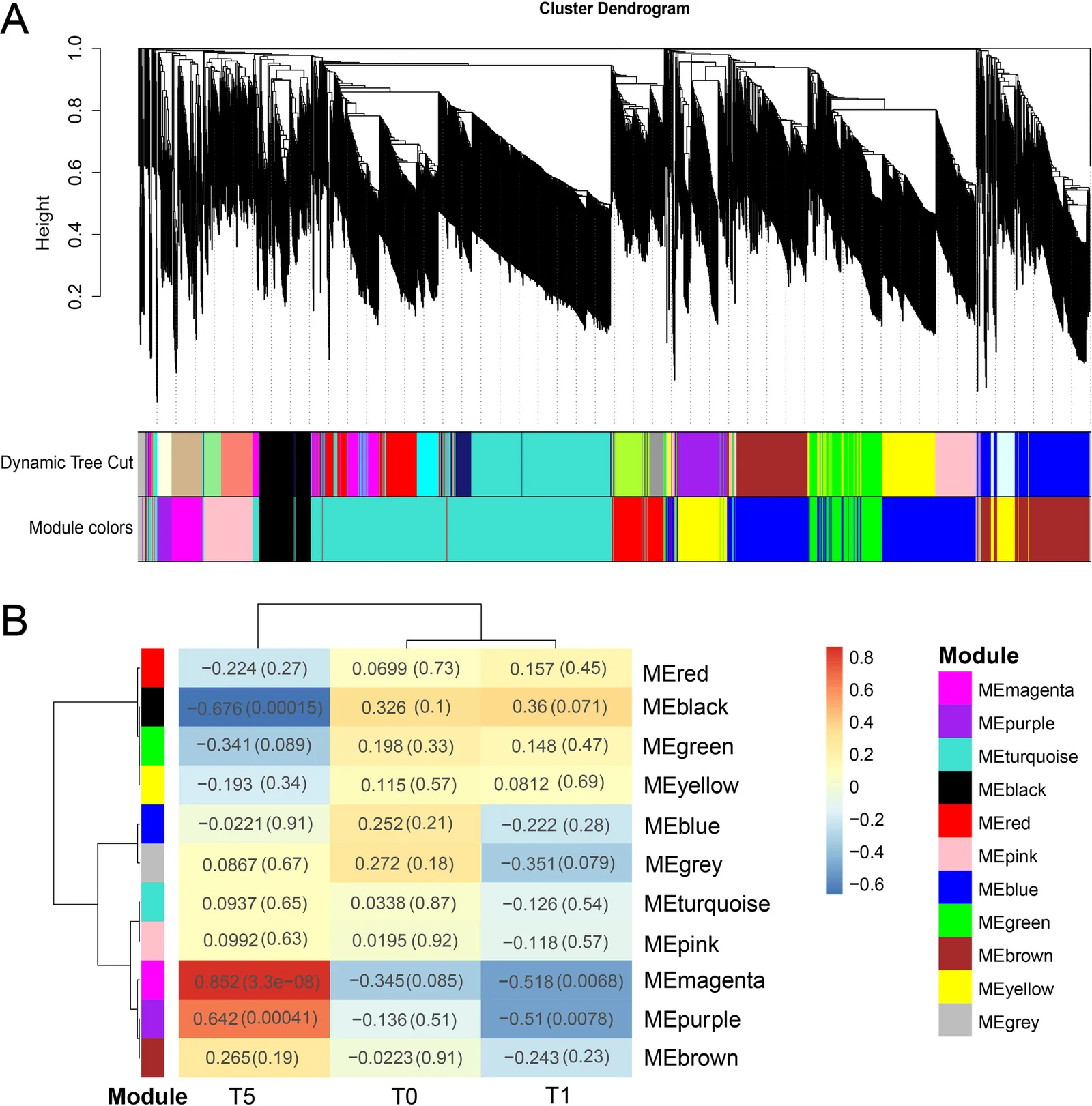
Weighted gene co‑expression network analysis (WGCNA) of the transcriptome in three examined groups (T0, T1 and T5) of *Andrias davidianus*. Notes: (**A**) Cluster dendrogram of gene modules. (**B**) Correlation heatmap between 11 identified modules and the three examined groups

In the analysis, a total of 11 core modules were identified (Fig. 5A). To identify the core modules highly associated with the three examined groups, we calculated the correlation (bicor) and corresponding p-values between the Module Eigengene (ME) of each module and the three examined groups (Fig. 5B). Module–trait correlation analysis revealed that the MEmagenta module exhibited a strong positive correlation with T5 (bicor = 0.852, *P* < 0.05) and a significant negative correlation with T1 (bicor = − 0.518, *P* < 0.05). Similarly, the MEpurple module showed a relatively strong positive correlation with T5 (bicor = 0.642, *P* < 0.05) and a significant negative correlation with T1 (bicor = − 0.510, *P* < 0.05). In contrast, the MEblack module displayed a significant negative correlation with T5 (bicor = − 0.676, *P* < 0.05). Other modules exhibited weaker correlations (|bicor| < 0.4) with the examined groups.

Notably, the MEmagenta module—which exhibited the strongest association with the elevated-temperature infection at the T5 stage—contained three *AndHSP* genes: *AndHSP20-01*, *AndHSP70-08*, and *AndHSP70-18*. Other modules, such as MEturquoise, MEpink, MEblue, MEblack, and MEbrown, also harbored subsets of *AndHSP* genes (Table 2). Within the significantly correlated MEmagenta module, 12 hub genes were identified (Supplementary Table 5), including *COL1A1* (encoding collagen alpha-1(I) chain), which is involved in extracellular matrix (ECM) remodeling and tissue repair.

**Table 2:** *AndHSPs* identified in different ME modules

Module name	Gene ID
MEturquoise	*AndHSP60-01*,* AndHSP40-07*,* AndHSP60-03*,* AndHSP60-04*,* AndHSP60-05*
MEmagenta	*AndHSP20-01*,* AndHSP70-08*,* AndHSP70-18*
MEpink	*AndHSP90-01*,* AndHSP70-16*,* AndHSP90-06*
MEblue	*AndHSP70-03*,* AndHSP60-06*
MEblack	*AndHSP70-11*,* AndHSP70-15*
MEbrown	*AndHSP90-02*

## Discussion

### Significance of identifying HSPs in *A. davidianus* and analysis of their basic physicochemical properties

As crucial stress-regulatory factors found across diverse organisms, HSPs play a central role in responses to both abiotic stresses (e.g., temperature, hunger, hypoxia) and biotic stresses (e.g., pathogen infection). They act as “molecular chaperones,” ensuring protein stability and function during stress conditions [54–58]. Since Ritossa’s discovery of HSPs in *Drosophila melanogaster* Meigen, 1830 in 1962 [59], substantial progress has been made in understanding their molecular functions and regulatory mechanisms [11, 12]. Although the roles of specific HSP genes and subfamilies are well-established in many plants and animals, studies that combine gene family identification with expression regulation under stress, particularly in non-model species, remain limited [55].

For the endangered Chinese giant salamander (*A. davidianus*), existing research has primarily focused on cloning and preliminary expression analyses of individual HSP genes [29, 35]. The genome-level identification and systematic analysis of HSP expression patterns, as presented in this study, marks a significant contribution to understanding the evolutionary relationships of HSP gene families in amphibians. In total, 72 *AndHSPs* genes were identified in *A. davidianus*, which is comparable to other amphibians, such as *X. tropicalis* (75) and *M. unicolor* (75). However, according to limited information of known HSP gene families in other animals, it is lower than that of species like the yellow fever mosquito (*Aedes aegypti* (Linnaeus, 1762), 80) [44]. In contrast, it is significantly higher than species like the Asian long-horned beetle (*Anoplophora glabripennis* (Motschulsky, 1854), 47) [49], the golden pompano (*Trachinotus ovatus* (Linnaeus, 1758), 43) [42], and the Chinese mitten crab (*Eriocheir sinensis* H. Milne Edwards, 1853, 56) [52]. These findings, even though from comparisons of some distantly related taxa, suggest that the size and distribution of HSP gene families are not solely determined by genome size but may also be influenced by a species’ genetic background and evolutionary adaptation strategies.

Furthermore, the study predicted that the overall isoelectric point (pI) of *AndHSPs* is predominantly acidic, a feature also noted in other species such as *A. glabripennis* [49], *T. ovatus* [42], and the green mirid bug (*Apolygus lucorum* (Meyer-Dür, 1843)) [53]. This acidic electrostatic surface property may facilitate interactions between HSPs and intracellular cations (e.g., Ca²⁺, Mg²⁺) or positively charged biomolecules (e.g., histones or receptors), which is critical for their chaperone function.

### Systematic evolution of HSPs in *A. davidianus* and the functional relevance of their structural characteristics

Phylogenetic analysis revealed that the *AndHSPs* clustered closely with those of *X. tropicalis* and *M. unicolor*, with all subfamilies present in all three species. This suggests that the HSP gene family has been highly conserved in amphibians, with its core biological functions possibly retained. Further analysis revealed that one branch of the HSP70 subfamily, HSP70-I, is more closely related to HSP40 and HSP90 than to HSP60, which aligns with known molecular functions: HSP40 acts as a co-chaperone to activate HSP70’s ATPase activity, and HSP70 collaborates with HSP90 to complete the maturation and stabilization of client proteins [11, 12, 44]. The phylogenetic proximity of *HSP70-I* with these subfamilies likely reflects evolutionary constraints imposed by their functional collaboration in protein folding. However, the relatively low bootstrap support for this relationship (< 80) suggests that further validation is required, particularly with the inclusion of more amphibian species.

Regarding gene family expansion, this study found that the *AndHSP40* subfamily had the highest number of members, while *AndHSP60* and *AndHSP90* were relatively smaller, though both also showed signs of expansion. For instance, while *X. tropicalis* and *M. unicolor* possess only one HSP60 gene, seven *AndHSP60* genes were identified in *A. davidianus*. A similar expansion pattern of HSP60 genes was also observed in *T. ovatus* [42]. Gene expansion could be driven by factors such as subfunctionalization, neofunctionalization [60]. This is supported by the distinct expression patterns observed within the *AndHSP40* subfamily; for instance, orthologs *AndHSP40-10*, *AndHSP40-28*, and *AndHSP40-29* exhibited divergent expression profiles across tissues and time points, suggesting possible functional divergence. Additionally, adaptation to diverse environments, improved sensory perception, or more efficient pathogen responses may contribute to this expansion. These hypotheses warrant further functional validation.

Gene structure, conserved motifs, and protein domains provided strong evidence for the classification of HSP subfamilies in *A. davidianus*. Members of the same subfamily exhibited high similarity in gene structure, motif composition, and core domains, supporting a common evolutionary origin and suggesting functional similarities [44]. The *AndHSP70* subfamily, for example, exhibited the most complex gene structure and the highest number of motifs, likely reflecting its involvement in diverse protein interactions during protein folding. Although some sequence information might have been missed due to transcriptome assembly limitations, the complexity of *AndHSP70*’s structure points to its multifaceted functional roles. The presence of specific domains is directly linked to the core functions of each subfamily. For instance, all members of the *AndHSP20* subfamily contain the signature alpha-crystallin domain, crucial for small HSPs in preventing protein misfolding under stress conditions [61]. The *AndHSP40* subfamily shows functional diversity due to varying domain compositions, including the typical DnaJ domain and other unique domains that may allow *AndHSP40* to participate in broader cellular functions, such as tRNA modification [62] and signal transduction [63]. Similarly, all *AndHSP90* members contain the conserved HSP90 domain [64], and the additional HATPase_Hsp90-like domain in *AndHSP90-01* suggests a unique regulatory function. These domains likely place *AndHSP90* as a key player in complex cellular networks, similar to *AndHSP70-06*, which also contains multiple domains.

Protein function predictions revealed that some *AndHSP40* and *AndHSP70* subfamily members possess transmembrane domains and signal peptides, as well as abundant glycosylation sites. These features are crucial for protein targeting, localization, and post-translational modification, all of which suggest that *AndHSP40* and *AndHSP70* are likely involved in membrane-related regions, such as the endoplasmic reticulum [65]. Subcellular localization predictions further confirmed that *AndHSPs* are distributed across multiple cellular compartments, including the cytoplasm, nucleus, and mitochondria. This diversity in localization supports their involvement in different cellular processes, consistent with findings in other species, such as *E. sinensis* [52]. Additionally, the diverse tertiary structural models of *AndHSP70* and *AndHSP90*, coupled with the extensive connectivity observed in the protein-protein interaction network, underscore the hub-like roles of these proteins within the cell. This collaborative network suggests that *AndHSPs*, especially *AndHSP70* and *AndHSP90*, likely cooperate across various cellular compartments to manage stress responses.

### Expression patterns of HSPs in *A. davidianus* under disease and temperature stress

Over long-term evolution, *A. davidianus* has developed robust immune defense mechanisms, with HSPs playing a pivotal role in resisting pathogen invasion [66]. Gene expression analysis revealed distinct stress response patterns: pathogen infection consistently induced the expression of *AndHSPs*, and elevated temperature treatment significantly intensified this response. Specifically, compared to normal temperature infection, the elevated temperature infection led to a higher number of DEGs at each time point, along with a more rapid response (Fig. 3B). Notably, nine *AndHSP* genes exhibited highly significant differential expression under elevated temperature infection, and their expression patterns correlated well with qPCR validation (Fig. 3C), indicating a coordinated stress response. However, the use of β-actin as the sole reference gene in the qPCR validation may limit the accuracy of gene quantification. Future studies could improve validation by incorporating multiple reference genes, such as *GAPDH* and *EF1a*.

Among the genes identified, *AndHSP70-18* exhibited the most dramatic upregulation as early as the initial stages of infection (T5), consistent with the behavior of a typical heat-inducible *HSP70* gene [67, 68]. This suggests that *AndHSP70-18* may play a pioneering role in initiating the stress response in the Chinese giant salamander. Further analysis of the multi-tissue transcriptome data revealed that all 72 *AndHSPs* genes were widely expressed across the kidney, liver, and spleen, though their expression patterns varied significantly between tissues. A subset of genes exhibited stable expression, indicating that these genes likely function as constitutive HSPs involved in fundamental physiological processes, such as maintaining protein homeostasis [69] or participating in cytoskeleton regulation, like HSP20 [70]. In contrast, another subset demonstrated strong inducible expression characteristics. The nine tissue-co-expressed DEGs identified in this study fall into this category, as they are more directly involved in protein folding, signal transduction, and immune responses under environmental stress.

An intriguing finding was that most *AndHSP60* genes had relatively high basal expression levels in all three tissues, with particularly significant expression in the spleen. This pattern is consistent with observations in the *T. ovatus* [42]. Given the spleen’s role as an important immune organ, this suggests that *AndHSP60* may have a specialized function in immune regulation in *A. davidianus*. However, its precise mechanism remains unclear, and further studies using techniques like RNA interference or gene editing are needed to fully elucidate its role in immune responses.

Finally, the temperature stress response was further validated by WGCNA, which identified three HSP genes (two *AndHSP70* and one *AndHSP20*) in the MEmagenta module as key participants in the early phases of temperature stress. Their upregulation corroborates the classic heat-inducible expression patterns widely reported in HSP families [71]. This result aligns with findings from other species, such as the Pacific oyster *Crassostrea gigasunder* (Thunberg, 1793) thermal stress [72]. Notably, the identification of the ECM-related hub gene *COL1A1* implies that the structural reinforcement of the cuticle may serve as a crucial defense strategy against the combined challenges of heat and pathogen stress.

## Conclusions

This study presents the first systematic identification and analysis of the HSP gene family in *A. davidianus* based on transcriptome data. A total of 72 *AndHSPs* genes were identified and classified into five subfamilies. The number and distribution of these genes reflect the evolutionary adaptation strategies of amphibian species. Phylogenetic and structural analyses revealed that each subfamily is evolutionarily conserved, with significant functional differentiation. The complex domain compositions of these proteins form the molecular basis for their functional diversity. Most importantly, by integrating multi-tissue expression data, this study unveiled the coordinated response of *AndHSPs* under the combined stresses of *A. hydrophila* infection and elevated temperature. Pathogen infection was identified as the core inducer, while elevated temperature significantly intensified this stress response. Nine key differentially expressed genes were highlighted, with *AndHSP70-18* exhibited rapid heat-inducible characteristics, and several *AndHSP60* members showed potential immunomodulatory roles, particularly in immune-related tissues such as the spleen. Overall, this study provides valuable insights into the composition, evolution, and functional roles of the HSP gene family in the environmental adaptation of this endangered amphibian species.

## Supplementary Information

Supplementary Material 1

## Data Availability

The transcriptome datasets of different tissues and under different stresses of *Andrias davidianus* are under the accession number in NCBI-Bioproject PRJNA1394661.
