# Supplementary material for: Identification and characterization of the HSP gene family in the Chinese giant salamander: Expression patterns under combined environmental stress
Source: BMC Genomics. 2026 Jun 24;27:743. doi: 10.1186/s12864-026-13091-1 (PMC13551934; doi:10.1186/s12864-026-13091-1)
Supplement: Supplementary file 1 [file 12864_2026_13091_MOESM1_ESM.docx]

**Supplementary Table 1**. Characteristics of the HSP gene family in *Andrais davidianus.*

| Sequence Names | Number of amino acids | Molecular weight (kDa) | Theoretical pI | Number of Negatively Charged Residues | Number of Positively Charged Residues | Amino Acid composition | N-terminal Amino Acid | Estimated half-life | Instability index | Aliphatic index | Grand average of hydropathicity |
| --- | --- | --- | --- | --- | --- | --- | --- | --- | --- | --- | --- |
| AndHSP20-01 | 171 | 19.54 | 5.89 | 25 | 20 | 20 | Met | 10095 | 55.95 | 77.43 | -0.437 |
| AndHSP20-02 | 174 | 19.93 | 6.16 | 26 | 23 | 19 | Met | 15470 | 46.63 | 86.26 | -0.371 |
| AndHSP20-03 | 168 | 18.42 | 5.18 | 20 | 16 | 19 | Met | 12170 | 61.51 | 68.99 | -0.444 |
| AndHSP20-04 | 203 | 23.01 | 6.32 | 28 | 25 | 19 | Gln | 12045 | 47.47 | 81.63 | -0.528 |
| AndHSP20-05 | 143 | 15.75 | 6.69 | 19 | 18 | 20 | Pro | 10220 | 63.4 | 66.78 | -0.628 |
| AndHSP20-06 | 215 | 23.90 | 5.86 | 27 | 24 | 20 | Met | 39085 | 69.88 | 73.07 | -0.439 |
| AndHSP20-07 | 285 | 31.19 | 6.76 | 36 | 36 | 20 | Met | 21470 | 52.48 | 66.46 | -0.571 |
| AndHSP20-08 | 204 | 22.58 | 4.70 | 30 | 18 | 20 | Met | 31065 | 56.37 | 70.74 | -0.496 |
| AndHSP20-09 | 168 | 18.57 | 6.43 | 22 | 21 | 19 | Met | 2980 | 58.06 | 59.76 | -0.691 |
| AndHSP40-01 | 405 | 45.53 | 8.33 | 55 | 59 | 20 | Met | 28140 | 29.79 | 73.14 | -0.64 |
| AndHSP40-02 | 793 | 90.44 | 7.27 | 101 | 101 | 20 | Met | 149670 | 36.2 | 79.57 | -0.405 |
| AndHSP40-03 | 338 | 38.17 | 8.61 | 51 | 54 | 20 | Met | 24535 | 41.42 | 68.7 | -0.719 |
| AndHSP40-04 | 380 | 42.73 | 8.54 | 51 | 55 | 20 | Met | 31205 | 39.35 | 59.61 | -0.883 |
| AndHSP40-05 | 584 | 66.63 | 5.96 | 97 | 86 | 20 | Leu | 56435 | 48.05 | 82.47 | -0.629 |
| AndHSP40-06 | 316 | 35.17 | 8.91 | 52 | 56 | 20 | Met | 20065 | 47.16 | 76.17 | -0.849 |
| AndHSP40-07 | 399 | 44.95 | 6.90 | 62 | 61 | 20 | Met | 17015 | 33.11 | 71.28 | -0.743 |
| AndHSP40-08 | 223 | 26.13 | 6.70 | 33 | 31 | 19 | Met | 10555 | 42.41 | 35.07 | -0.953 |
| AndHSP40-09 | 768 | 85.23 | 8.83 | 97 | 113 | 19 | Met | 95600 | 52.16 | 62.16 | -0.677 |
| AndHSP40-10 | 327 | 36.74 | 8.94 | 47 | 52 | 20 | Met | 26025 | 58.5 | 37.89 | -1.037 |
| AndHSP40-11 | 2240 | 254.98 | 6.40 | 277 | 258 | 20 | Met | 241320 | 40.94 | 91.9 | -0.256 |
| AndHSP40-12 | 398 | 44.92 | 8.66 | 54 | 58 | 20 | Met | 30160 | 45.34 | 65.88 | -0.707 |
| AndHSP40-13 | 469 | 51.55 | 9.26 | 48 | 63 | 20 | Met | 39475 | 43.03 | 61.96 | -0.544 |
| AndHSP40-14 | 269 | 30.89 | 9.55 | 31 | 43 | 20 | Met | 33140 | 62.7 | 58.77 | -0.919 |
| AndHSP40-15 | 198 | 22.11 | 5.04 | 29 | 20 | 20 | Met | 26775 | 37.96 | 62.12 | -0.586 |
| AndHSP40-16 | 149 | 17.38 | 4.59 | 30 | 17 | 20 | Met | 32805 | 54.21 | 71.34 | -0.668 |
| AndHSP40-17 | 185 | 20.97 | 5.98 | 30 | 28 | 20 | Met | 21275 | 38.48 | 56.43 | -0.779 |
| AndHSP40-18 | 553 | 63.61 | 5.37 | 111 | 93 | 20 | Met | 60445 | 56.84 | 55.06 | -1.159 |
| AndHSP40-19 | 352 | 40.00 | 7.29 | 46 | 46 | 20 | Met | 29465 | 40.92 | 60.97 | -0.866 |
| AndHSP40-20 | 246 | 27.94 | 8.08 | 34 | 36 | 20 | Met | 30285 | 38.19 | 74.88 | -0.412 |
| AndHSP40-21 | 318 | 37.49 | 9.47 | 43 | 64 | 20 | Met | 60445 | 49.28 | 65.47 | -0.936 |
| AndHSP40-22 | 532 | 62.05 | 8.22 | 91 | 94 | 20 | Met | 86205 | 52.44 | 80.77 | -0.819 |
| AndHSP40-23 | 618 | 69.64 | 8.78 | 75 | 82 | 20 | Met | 63175 | 47.32 | 84.56 | -0.355 |
| AndHSP40-24 | 374 | 41.71 | 6.58 | 53 | 51 | 20 | Met | 21930 | 37.83 | 77.3 | -0.526 |
| AndHSP40-25 | 154 | 16.60 | 9.86 | 14 | 23 | 20 | Met | 14440 | 50.34 | 74.94 | -0.472 |
| AndHSP40-26 | 482 | 54.60 | 8.87 | 61 | 70 | 20 | Met | 55070 | 60.12 | 57.18 | -0.835 |
| AndHSP40-27 | 379 | 43.98 | 9.16 | 47 | 57 | 20 | Met | 44600 | 34.64 | 74.33 | -0.681 |
| AndHSP40-28 | 468 | 51.56 | 9.52 | 51 | 65 | 20 | Met | 48610 | 50.56 | 73.42 | -0.594 |
| AndHSP40-29 | 468 | 51.56 | 9.52 | 51 | 65 | 20 | Met | 48610 | 50.56 | 73.42 | -0.594 |
| AndHSP60-01 | 535 | 57.85 | 5.86 | 74 | 63 | 20 | Met | 13325 | 28.3 | 102 | -0.024 |
| AndHSP60-02 | 237 | 25.37 | 9.52 | 28 | 35 | 20 | Met | 13980 | 38.81 | 97.51 | -0.199 |
| AndHSP60-03 | 541 | 59.62 | 5.28 | 81 | 66 | 20 | Met | 20440 | 38.67 | 97.74 | -0.164 |
| AndHSP60-04 | 487 | 53.38 | 6.25 | 63 | 60 | 20 | Met | 19410 | 38.04 | 103.18 | -0.044 |
| AndHSP60-05 | 537 | 57.82 | 8.19 | 63 | 66 | 20 | Met | 12545 | 34.89 | 108.58 | 0.036 |
| AndHSP60-06 | 576 | 61.28 | 5.57 | 80 | 73 | 20 | Met | 14565 | 27.26 | 102.59 | -0.039 |
| AndHSP60-07 | 115 | 12.04 | 4.57 | 17 | 11 | 17 | Leu | 1490 | 34.06 | 106.87 | 0.183 |
| AndHSP70-01 | 402 | 45.11 | 5.57 | 66 | 59 | 20 | Ile | 17545 | 32.99 | 83.71 | -0.628 |
| AndHSP70-02 | 177 | 19.39 | 4.96 | 24 | 18 | 19 | Ser | 4595 | 31.61 | 97.01 | -0.11 |
| AndHSP70-03 | 680 | 73.36 | 5.53 | 94 | 85 | 20 | Met | 29255 | 42.27 | 83.94 | -0.367 |
| AndHSP70-04 | 681 | 76.27 | 8.77 | 78 | 87 | 20 | Met | 65610 | 45.77 | 80.91 | -0.31 |
| AndHSP70-05 | 785 | 88.46 | 5.14 | 128 | 104 | 20 | Met | 55780 | 40.86 | 76.62 | -0.55 |
| AndHSP70-06 | 983 | 109.97 | 5.14 | 172 | 139 | 20 | Met | 57550 | 42.29 | 81.37 | -0.577 |
| AndHSP70-07 | 484 | 53.43 | 6.62 | 51 | 49 | 20 | Met | 33350 | 36.89 | 94.44 | -0.168 |
| AndHSP70-08 | 655 | 72.58 | 5.06 | 89 | 112 | 20 | Met | 30495 | 33.47 | 86.44 | -0.484 |
| AndHSP70-09 | 109 | 12.13 | 7.04 | 15 | 15 | 19 | Ile | 8480 | 32.94 | 67.06 | -0.642 |
| AndHSP70-10 | 125 | 13.90 | 6.81 | 18 | 18 | 18 | Ser | 4470 | 43.39 | 86.64 | -0.416 |
| AndHSP70-11 | 163 | 18.46 | 9.17 | 22 | 27 | 19 | Met | 4595 | 35.35 | 92.21 | -0.261 |
| AndHSP70-12 | 647 | 71.86 | 6.49 | 75 | 71 | 20 | Met | 71655 | 44.56 | 82.6 | -0.215 |
| AndHSP70-13 | 100 | 11.06 | 11.44 | 5 | 21 | 17 | Met | 5625 | 40.51 | 64.4 | -0.854 |
| AndHSP70-14 | 104 | 11.17 | 9.57 | 11 | 15 | 18 | Phe | 4470 | 45.85 | 81.73 | -0.292 |
| AndHSP70-15 | 173 | 19.07 | 5.92 | 20 | 18 | 20 | Met | 18700 | 36.56 | 79.94 | -0.129 |
| AndHSP70-16 | 142 | 16.28 | 5.37 | 29 | 25 | 20 | Asp | 11585 | 34.15 | 55.63 | -1.279 |
| AndHSP70-17 | 571 | 63.52 | 6.13 | 84 | 80 | 20 | Met | 33140 | 38.98 | 83.01 | -0.494 |
| AndHSP70-18 | 648 | 71.17 | 5.36 | 94 | 80 | 20 | Met | 35090 | 35.63 | 81.57 | -0.453 |
| AndHSP70-19 | 198 | 22.50 | 7.00 | 23 | 23 | 20 | Met | 27180 | 38.86 | 88.08 | -0.223 |
| AndHSP70-20 | 794 | 89.58 | 5.72 | 121 | 108 | 20 | Met | 55655 | 41.87 | 81.26 | -0.414 |
| AndHSP70-21 | 508 | 54.59 | 5.18 | 66 | 50 | 19 | Met | 15775 | 34.83 | 96.14 | -0.038 |
| AndHSP90-01 | 292 | 32.61 | 4.66 | 57 | 35 | 19 | Met | 17420 | 41.57 | 80.55 | -0.54 |
| AndHSP90-02 | 128 | 14.33 | 3.98 | 33 | 9 | 19 | Lys | 4595 | 50.14 | 90.7 | -0.502 |
| AndHSP90-03 | 327 | 37.28 | 4.56 | 74 | 43 | 20 | Met | 31400 | 43.24 | 75.2 | -0.805 |
| AndHSP90-04 | 103 | 12.10 | 5.16 | 31 | 25 | 17 | Asp | 5960 | 53.55 | 47.28 | -1.868 |
| AndHSP90-05 | 120 | 13.85 | 8.84 | 18 | 21 | 20 | Met | 17085 | 40.27 | 65.08 | -0.826 |
| AndHSP90-06 | 259 | 29.59 | 4.82 | 52 | 36 | 20 | Met | 17670 | 40.11 | 79.73 | -0.566 |

**Supplementary Table 2**. Orthogroup distribution of the heat shock protein gene family across three species

| Orthogroup | AndHSP | MicHSP | XenHSP |
| --- | --- | --- | --- |
| OG0000000 | AndHSP90-01, AndHSP90-02, AndHSP90-03, AndHSP90-04, AndHSP90-05, AndHSP90-06 | MicHSP90-01, MicHSP90-02, MicHSP90AA1, MicHSP90AB1, MicHSP90B1 | XenHSP90-01, XenHSP90aa1.1, XenHSP90aa1.2, XenHSP90ab1, XenHSP90b1 |
| OG0000001 | AndHSP70-01, AndHSP70-02, AndHSP70-03, AndHSP70-08, AndHSP70-09, AndHSP70-10, AndHSP70-11, AndHSP70-13, AndHSP70-14, AndHSP70-15, AndHSP70-16, AndHSP70-17, AndHSP70-18 |  |  |
| OG0000002 | AndHSP40-01, AndHSP40-07 | MicHSP40-01, MicHSP40-03, MicHSP40A1, MicHSP40A2 | XenHSP40a1, XenHSP40a2, XenHSP40a4.1, XenHSP40a4.2, XenHSP40c16 |
| OG0000003 | AndHSP40-04, AndHSP40-19, AndHSP40-26 | MicHSP40-04, MicHSP40B12, MicHSP40B14 | XenHSP40b12, XenHSP40b14, XenHSP40c18 |
| OG0000004 |  | MicHSP70A5, MicHSP70A9 | XenHSP70-01, XenHSP70a1a, XenHSP70a1l, XenHSP70a2, XenHSP70a5, XenHSP70a8, XenHSP70a9 |
| OG0000005 | AndHSP40-03, AndHSP40-12 | MicHSP40B1, MicHSP40B4, MicHSP40B5 | XenHSP40b1, XenHSP40b4, XenHSP40b5 |
| OG0000006 |  | MicHSP70-02, MicHSP70-03, MicHSP70-04, MicHSP70-05, MicHSP70-06, MicHSP70-07, MicHSP70A2, MicHSP70A8 |  |
| OG0000007 | AndHSP40-15, AndHSP40-17 | MicHSP40C5, MicHSP40C5G | XenHSP40c5, XenHSP40c5g |
| OG0000008 | AndHSP70-04, AndHSP70-12, AndHSP70-19 | MicHSP70A12B | XenHSP70a12a, XenHSP70a12b |
| OG0000009 | AndHSP70-05, AndHSP70-20 | MicHSP70A4, MicHSP70H1 | XenHSP70a4, XenHSP70h1 |
| OG0000010 | AndHSP20-01, AndHSP20-02 | MicHSP20B6 | XenHSP20-02, XenHSP20b6 |
| OG0000011 | AndHSP60-02, AndHSP60-06, AndHSP60-07 | MicHSP60D1 | XenHSP60d1 |
| OG0000012 |  |  | XenHSP20-03, XenHSP20-04, XensHSP20c, XensHSP20d, XensHSP20e |
| OG0000013 | AndHSP20-03, AndHSP20-09 | MicHSP20B7 | XenHSP20b7 |
| OG0000014 | AndHSP20-07 | MicHSP20-05, MicHSP20-06 | XenHSP20-05 |
| OG0000015 | AndHSP40-05 | MicHSP40-02, MicHSP40C3 | XenHSP40c3 |
| OG0000016 | AndHSP40-06 | MicHSP40-05, MicHSP40C17 | XenHSP40c17 |
| OG0000017 | AndHSP40-10 | MicHSP40B6 | XenHSP40b2, XenHSP40b6 |
| OG0000018 | AndHSP40-28, AndHSP40-29 | MicHSP40C30 | XenHSP40c30 |
| OG0000019 | AndHSP60-01, AndHSP60-03, AndHSP60-04, AndHSP60-05 |  |  |
| OG0000020 |  | MicHSP20-01, MicHSP20-02, MicHSP20-03, MicHSP20-04 |  |
| OG0000021 | AndHSP20-06 | MicHSP20B1 | XenHSP20b1 |
| OG0000022 | AndHSP20-08 | MicHSP20B8 | XenHSP20b8 |
| OG0000023 | AndHSP40-08 | MicHSP40B9 | XenHSP40b9 |
| OG0000024 | AndHSP40-09 | MicHSP40C14 | XenHSP40c14 |
| OG0000025 | AndHSP40-11 | MicHSP40C13 | XenHSP40c13 |
| OG0000026 | AndHSP40-13 | MicHSP40A3 | XenHSP40a3 |
| OG0000027 | AndHSP40-14 | MicHSP40C4 | XenHSP40c4 |
| OG0000028 | AndHSP40-16 | MicHSP40C24 | XenHSP40c24 |
| OG0000029 | AndHSP40-18 | MicHSP40C21 | XenHSP40c21 |
| OG0000030 | AndHSP40-21 | MicHSP40C7 | XenHSP40c7 |
| OG0000031 | AndHSP40-22 | MicHSP40C1 | XenHSP40c1 |
| OG0000032 | AndHSP40-23 | MicHSP40C11 | XenHSP40c11 |
| OG0000033 | AndHSP40-25 | MicHSP40C15 | XenHSP40c15 |
| OG0000034 | AndHSP40-27 | MicHSP40C28 | XenHSP40c28 |
| OG0000035 | AndHSP70-06 | MicHSP70-01 | XenHSP70-02 |
| OG0000036 | AndHSP70-07 | MicHSP70A13 | XenHSP70a13 |
| OG0000037 | AndHSP70-21 | MicHSP70A14 | XenHSP70a14 |
| OG0000038 |  | MicHSP40-06, MicHSP40B13 | XenHSP40b13 |
| OG0000039 |  | MicHSP40C5B | XenHSP40c5b, XenHSP40c6 |
| OG0000040 | AndHSP20-04 |  | XenHSP20b2 |
| OG0000041 | AndHSP20-05 |  | XenHSP20-01 |
| OG0000042 | AndHSP40-02 | MicHSP40C10 |  |
| OG0000043 | AndHSP40-20 | MicHSP40C27 |  |
| OG0000044 | AndHSP40-24 |  | XenHSP40b11 |
| OG0000045 |  | MicHSP20B3 | XenHSP20b3 |
| OG0000046 |  | MicHSP40C12 | XenHSP40c12 |
| OG0000047 |  | MicHSP40C2 | XenHSP40c2 |
| OG0000048 |  | MicHSP40C22 | XenHSP40c22 |
| OG0000049 |  | MicHSP40C25 | XenHSP40c25 |
| OG0000050 |  | MicHSP40C8 | XenHSP40c8 |
| OG0000051 |  | MicHSP40C9 | XenHSP40c9 |

**Supplementary Table 3**. Prediction of transmembrane regions, glycosylation sites, and subcellular localization of the HSP gene family in *Andrais davidianus*

| Sequence name | Number of transmembrane regions | Signal peptide position | Number of glycosylation site | α-Helix | β-Turn | Random Coil | Extended Strand | Subcellular location |
| --- | --- | --- | --- | --- | --- | --- | --- | --- |
| AndHSP20-01 | 0 | 0 | 1 | 25.15% | 4.09% | 53.80% | 16.96% | mitochondria |
| AndHSP20-02 | 0 | 0 | 0 | 18.39% | 4.02% | 60.92% | 16.09% | mitochondria |
| AndHSP20-03 | 0 | 0 | 2 | 6.55% | 4.17% | 74.40% | 14.88% | nucleus |
| AndHSP20-04 | 0 | 0 | 1 | 15.27% | 2.96% | 65.52% | 16.26% | cytoplasm |
| AndHSP20-05 | 0 | 0 | 0 | 10.49% | 4.90% | 62.94% | 21.68% | extracellular space |
| AndHSP20-06 | 0 | 0 | 0 | 10.70% | 3.26% | 72.56% | 13.49% | nucleus |
| AndHSP20-07 | 0 | 0 | 1 | 25.61% | 2.81% | 61.40% | 10.18% | nucleus |
| AndHSP20-08 | 0 | 0 | 0 | 9.31% | 3.43% | 74.02% | 13.24% | mitochondria |
| AndHSP20-09 | 0 | 0 | 0 | 20.24% | 4.17% | 57.14% | 18.45% | nucleus |
| AndHSP40-01 | 0 | 0 | 0 | 19.75% | 6.91% | 56.79% | 18.02% | cytoplasm |
| AndHSP40-02 | 0 | 1-28 | 2 | 39.72% | 1.89% | 42.50% | 15.89% | extracellular space |
| AndHSP40-03 | 0 | 0 | 1 | 24.85% | 6.21% | 53.25% | 15.68% | nucleus |
| AndHSP40-04 | 0 | 0 | 1 | 42.63% | 1.58% | 50.00% | 5.79% | plasma membrane |
| AndHSP40-05 | 0 | 0 | 0 | 65.24% | 3.77% | 25.34% | 5.65% | cytoplasm |
| AndHSP40-06 | 0 | 0 | 0 | 59.18% | 0.63% | 31.96% | 8.23% | cytoplasm |
| AndHSP40-07 | 0 | 0 | 1 | 22.81% | 7.27% | 53.38% | 16.54% | cytoplasm |
| AndHSP40-08 | 0 | 1-23 | 0 | 26.91% | 0.90% | 69.51% | 2.69% | extracellular space |
| AndHSP40-09 | 0 | 0 | 3 | 33.98% | 2.47% | 58.46% | 5.08% | plasma membrane |
| AndHSP40-10 | 0 | 0 | 0 | 20.49% | 3.36% | 68.20% | 7.95% | nucleus |
| AndHSP40-11 | 0 | 0 | 8 | 60.85% | 2.01% | 29.69% | 7.46% | nucleus |
| AndHSP40-12 | 0 | 0 | 1 | 21.86% | 6.03% | 56.78% | 15.33% | cytoplasm |
| AndHSP40-13 | 0 | 0 | 4 | 20.68% | 3.84% | 59.28% | 16.20% | mitochondria |
| AndHSP40-14 | 2 | 0 | 2 | 42.01% | 1.12% | 51.67% | 5.20% | extracellular space |
| AndHSP40-15 | 2 | 0 | 1 | 29.80% | 2.02% | 62.63% | 5.56% | extracellular space |
| AndHSP40-16 | 0 | 0 | 0 | 40.27% | 2.68% | 40.27% | 16.78% | extracellular space |
| AndHSP40-17 | 0 | 0 | 0 | 32.97% | 1.08% | 58.92% | 7.03% | extracellular space |
| AndHSP40-18 | 0 | 0 | 0 | 58.59% | 1.27% | 37.43% | 2.71% | nucleus |
| AndHSP40-19 | 1 | 0 | 1 | 43.47% | 1.14% | 48.86% | 6.53% | plasma membrane |
| AndHSP40-20 | 0 | 0 | 0 | 41.46% | 5.69% | 35.77% | 17.07% | cytoplasm |
| AndHSP40-21 | 0 | 0 | 0 | 67.30% | 2.20% | 26.73% | 3.77% | cytoplasm |
| AndHSP40-22 | 1 | 1-22 | 3 | 52.26% | 1.13% | 44.36% | 2.26% | endoplasmic reticulum |
| AndHSP40-23 | 0 | 0 | 2 | 35.92% | 2.10% | 46.60% | 15.37% | plasma membrane |
| AndHSP40-24 | 0 | 0 | 1 | 27.54% | 7.22% | 48.66% | 16.58% | mitochondria |
| AndHSP40-25 | 0 | 0 | 0 | 54.55% | 0.00% | 41.56% | 3.90% | mitochondria |
| AndHSP40-26 | 1 | 0 | 1 | 39.42% | 1.04% | 54.56% | 4.98% | plasma membrane |
| AndHSP40-27 | 0 | 0 | 4 | 61.74% | 1.32% | 34.56% | 2.37% | cytoplasm |
| AndHSP40-28 | 1 | 0 | 1 | 32.69% | 5.98% | 55.56% | 5.77% | plasma membrane |
| AndHSP40-29 | 1 | 0 | 1 | 32.69% | 5.98% | 55.56% | 5.77% | plasma membrane |
| AndHSP60-01 | 0 | 0 | 2 | 53.46% | 6.17% | 26.36% | 14.02% | cytoplasm |
| AndHSP60-02 | 0 | 0 | 0 | 52.74% | 7.59% | 29.96% | 9.70% | cytoplasm |
| AndHSP60-03 | 0 | 0 | 0 | 53.60% | 6.84% | 25.32% | 14.23% | cytoplasm |
| AndHSP60-04 | 0 | 0 | 0 | 51.13% | 6.78% | 26.90% | 15.20% | cytoplasm |
| AndHSP60-05 | 0 | 0 | 3 | 54.00% | 6.70% | 26.07% | 13.22% | cytoplasm |
| AndHSP60-06 | 0 | 0 | 2 | 55.73% | 5.73% | 27.95% | 10.59% | mitochondria |
| AndHSP60-07 | 0 | 0 | 0 | 61.74% | 5.22% | 31.30% | 1.74% | Cytoplasm,nucleus |
| AndHSP70-01 | 0 | 0 | 4 | 49.25% | 6.72% | 28.86% | 15.17% | Cytoplasm,nucleus |
| AndHSP70-02 | 0 | 0 | 2 | 38.98% | 10.17% | 33.33% | 17.51% | cytoskeleton |
| AndHSP70-03 | 0 | 0 | 3 | 41.47% | 6.32% | 33.09% | 19.12% | mitochondria |
| AndHSP70-04 | 0 | 0 | 2 | 28.63% | 5.58% | 45.81% | 19.97% | cytoplasm |
| AndHSP70-05 | 0 | 0 | 4 | 44.33% | 2.04% | 42.68% | 10.96% | nucleus |
| AndHSP70-06 | 0 | 1-25 | 8 | 46.08% | 2.85% | 40.18% | 10.89% | endoplasmic reticulum |
| AndHSP70-07 | 0 | 1-22 | 5 | 42.98% | 7.02% | 32.85% | 17.15% | endoplasmic reticulum |
| AndHSP70-08 | 0 | 1-20 | 0 | 43.66% | 6.87% | 31.15% | 18.32% | endoplasmic reticulum |
| AndHSP70-09 | 0 | 0 | 1 | 38.53% | 7.34% | 33.94% | 20.18% | cytoplasm |
| AndHSP70-10 | 0 | 0 | 1 | 34.40% | 12.00% | 28.80% | 24.80% | nucleus |
| AndHSP70-11 | 0 | 0 | 1 | 63.19% | 6.75% | 23.31% | 6.75% | cytoplasm,nucleus |
| AndHSP70-12 | 0 | 0 | 1 | 29.52% | 4.48% | 45.44% | 20.56% | cytoplasm |
| AndHSP70-13 | 0 | 0 | 1 | 38.00% | 6.00% | 34.00% | 22.00% | mitochondria |
| AndHSP70-14 | 0 | 0 | 0 | 49.04% | 3.85% | 26.92% | 20.19% | cytoplasm |
| AndHSP70-15 | 0 | 0 | 2 | 35.26% | 7.51% | 34.68% | 22.54% | cytoplasm |
| AndHSP70-16 | 0 | 0 | 0 | 70.42% | 1.41% | 24.65% | 3.52% | cytoplasm |
| AndHSP70-17 | 0 | 0 | 5 | 45.53% | 6.65% | 29.07% | 18.74% | Cytoplasm,nucleus |
| AndHSP70-18 | 0 | 0 | 7 | 41.82% | 6.02% | 33.33% | 18.83% | cytoplasm |
| AndHSP70-19 | 0 | 0 | 1 | 53.03% | 4.04% | 27.78% | 15.15% | cytoplasm |
| AndHSP70-20 | 0 | 0 | 4 | 46.10% | 2.52% | 37.41% | 13.98% | cytoplasm |
| AndHSP70-21 | 0 | 0 | 1 | 36.02% | 6.50% | 33.46% | 24.02% | plasma membrane |
| AndHSP90-01 | 0 | 0 | 1 | 38.70% | 4.79% | 43.49% | 13.01% | cytoplasm |
| AndHSP90-02 | 0 | 0 | 1 | 55.47% | 2.34% | 42.19% | 0.00% | extracellular space |
| AndHSP90-03 | 0 | 0 | 2 | 45.87% | 4.89% | 34.25% | 14.98% | cytoplasm |
| AndHSP90-04 | 0 | 0 | 1 | 70.87% | 0.00% | 23.30% | 5.83% | cytoplasm |
| AndHSP90-05 | 0 | 0 | 0 | 50.83% | 3.33% | 30.83% | 15.00% | cytoplasm |
| AndHSP90-06 | 0 | 0 | 1 | 53.67% | 3.86% | 33.20% | 9.27% | cytoplasm |

Supplementary Figure 1. BH Test Values for Phylogenetic Relationships of Heat Shock Proteins in *Andrias davidianus*, *Xenopus tropicalis*, and *Microcaecilia unicolor*.

| AndHSP40-14 | AndHSP40-15 | AndHSP40-19 | AndHSP40-22 |
| --- | --- | --- | --- |
| AndHSP40-26 | AndHSP40-28 | AndHSP40-29 |  |

**Supplementary Figure 2**. Prediction of transmembrane regions of the HSPs in *Andrias davidianus*.

***Notes***: The transmembrane regions are indicated by red lines, the inner side of the cell membrane by yellow lines, and the outer side of the cell membrane by blue areas.

|   AndHSP40-02 |   AndHSP40-08 |   AndHSP40-22 |
| --- | --- | --- |
|   AndHSP70-06 |   AndHSP70-07 |   AndHSP70-08 |

**Supplementary Figure 3**. Prediction of signal peptides of the HSPs in *Andrias davidianus*.

***Notes***: Sec/SPI n denotes the N-terminal positively charged region of the signal peptide, Sec/SPI h represents the central hydrophobic core segment, and Sec/SPI c corresponds to the C-terminal region near the cleavage site of the signal peptide.

|  |  |  |  |  |  |  |
| --- | --- | --- | --- | --- | --- | --- |
| AndHSP20-01 | AndHSP20-02 | AndHSP20-05 | AndHSP60-01 | AndHSP60-02 | AndHSP60-03 | AndHSP60-04 |
|  |  |  |  |  |  |  |
| AndHSP60-05 | AndHSP60-06 | AndHSP60-07 | AndHSP70-01 | AndHSP70-02 | AndHSP70-07 | AndHSP70-08 |
|  |  |  |  |  |  |  |
| AndHSP70-09 | AndHSP70-10 | AndHSP70-11 | AndHSP70-13 | AndHSP70-14 | AndHSP70-15 | AndHSP70-17 |
|  |  |  |  |  |  |  |
| AndHSP70-18 | AndHSP70-19 | AndHSP70-21 | AndHSP90-03 | AndHSP90-05 | AndHSP90-06 |  |

**Supplementary Figure 4**. Three-dimensional structure prediction of some HSPs in *Andrias davidianus*.

**Supplementary Figure 5**. Protein-protein interaction network of HSPs in *Andrias davidianus*, with the size and color of the circle represent interaction degree.

**Supplementary Table 4**. Number of differentially expressed *AndHSPs* genes in different comparison groups based on DESeq2

| Tissue | Time | T1 | T2 | T3 | T4 | T5 | T6 | T7 | T8 |
| --- | --- | --- | --- | --- | --- | --- | --- | --- | --- |
| Kidney | T0 | 0 | 1 | 4 | 19 | 10 | 10 | 13 | 25 |
|  | T1 |  | 1 |  |  | 12 |  |  |  |
|  | T2 |  |  | 1 |  |  | 10 |  |  |
|  | T3 |  |  |  | 20 |  |  | 10 |  |
|  | T4 |  |  |  |  |  |  |  | 6 |
|  | T5 |  |  |  |  |  | 4 |  |  |
|  | T6 |  |  |  |  |  |  | 4 |  |
|  | T7 |  |  |  |  |  |  |  | 8 |
|  | T8 |  |  |  |  |  |  |  |  |
| Liver | T0 | 2 | 3 | 12 | 27 | 21 | 19 | 16 | 29 |
|  | T1 |  | 4 |  |  | 16 |  |  |  |
|  | T2 |  |  | 10 |  |  | 15 |  |  |
|  | T3 |  |  |  | 21 |  |  | 17 |  |
|  | T4 |  |  |  |  |  |  |  | 10 |
|  | T5 |  |  |  |  |  | 5 |  |  |
|  | T6 |  |  |  |  |  |  | 5 |  |
|  | T7 |  |  |  |  |  |  |  | 10 |
|  | T8 |  |  |  |  |  |  |  |  |
| Spleen | T0 | 1 | 2 | 2 | 28 | 17 | 14 | 12 | 29 |
|  | T1 |  | 2 |  |  | 13 |  |  |  |
|  | T2 |  |  | 1 |  |  | 13 |  |  |
|  | T3 |  |  |  | 28 |  |  | 13 |  |
|  | T4 |  |  |  |  |  |  |  | 4 |
|  | T5 |  |  |  |  |  | 3 |  |  |
|  | T6 |  |  |  |  |  |  | 5 |  |
|  | T7 |  |  |  |  |  |  |  | 10 |
|  | T8 |  |  |  |  |  |  |  |  |

**Supplementary Figure 6**. Heat map showing the expression of the five *AndHSPs* subfamilies under normal and elevated temperature infections. (A) *AndHSP20*. (B) *AndHSP40*. (C) *AndHSP60*. (D) *AndHSP70*. (E) *AndHSP90*.

**Supplementary Table 5**. List of hub genes in the MEmagenta

| Hub genes | Weight | Gene annotation |
| --- | --- | --- |
| TRINITY_DN30679_c1_g1 | 4.94 | \ |
| TRINITY_DN2580_c1_g1 | 4.93 | \ |
| TRINITY_DN107015_c0_g2 | 4.75 | \ |
| TRINITY_DN7029_c0_g1 | 4.62 | ERVFRD-1 |
| TRINITY_DN118336_c0_g1 | 4.4 |  |
| TRINITY_DN49800_c0_g1 | 4.06 |  |
| TRINITY_DN43097_c1_g1 | 4.01 |  |
| TRINITY_DN43097_c0_g1 | 3.97 |  |
| TRINITY_DN2006_c0_g2 | 2.76 |  |
| TRINITY_DN352267_c0_g1 | 1.11 |  |
| TRINITY_DN1909_c2_g5 | 1.09 |  |
| TRINITY_DN4676_c0_g2 | 1.08 | COL1A1 |
